# Systematic assessment of intrinsic factors influencing visual attention performances in air traffic control via clustering algorithm and statistical inference

**DOI:** 10.1371/journal.pone.0205334

**Published:** 2018-10-25

**Authors:** Jing-Qiang Li, Hong-Yan Zhang, Yan Zhang, Hai-Tao Liu

**Affiliations:** 1 Research Institute of Civil Aviation Safety, Civil Aviation University of China, Tianjin 300300, P. R. China; 2 National Key Laboratory of Air Traffic Operation Safety Technology, Civil Aviation University of China, Tianjin 300300, P. R. China; 3 Sino-European Institute of Aviation Engineering, Civil Aviation University of China, Tianjin 300300, P. R. China; 4 College of Electronic Information and Automation, Civil Aviation University of China, Tianjin 300300, P. R. China; Southwest University, CHINA

## Abstract

The intrinsic factors (IF) influencing visual attention performance (VAP) might cause potential human errors, such as “error/mistake”, “forgetting” and “omission”. It is a key issue to develop a systematic assessment of IF in order to distinguish the levels of VAP. Motivated by the Stimulus-Response (S-R) model, we take an interactive cancellation test—Neuron Type Test (NTT)—to explore the IF and present the corresponding systematic assessment. The main contributions of this work include three elements: a) modeling the IF on account of attention span, attention stability, distribution-shift of attention with measurable parameters by combining the psychological and statistical concepts; b) proposing quantitative analysis methods for assessing the IF via its computational representation—intrinsic qualities (IQ)—in the sense of computational model; and c) clustering the IQ of air traffic control (ATC) students in the feature space of interest. The response sequences of participants collected with the NTT system are characterized by three parameters: Hurst exponent, normalized number of decisions (NNoD) and error rate of decisions (ERD). The *K*-means clustering is applied to partition the feature space constructed from practical data of VAP. For the distinguishable clusters, the statistical inference is utilized to refine the assessment of IF. Our comprehensive analysis shows that the IQ can be classified into four levels, i.e., *excellent, good, moderate* and *unqualified*, which has a potential application in selecting air traffic controllers subject to reducing the risk of the inadequacy of attention performances in aviation safety management.

## Nomenclature

For the convience of writing and reading, we summarize the abbreviations and the corresponding full names adopted in this paper in Table 1.

**Table 1:** Nomenclature: Abbreviations vs. full names.

Abbr.	Full Name	Abbr.	Full Name
ATC	Air Traffic Control	NoD	Number of Decisions
ASRS	Aviation Safety Reporting System	NNoD	Normalized NoD
EF	Extrinsic Factors	NTT	Neuron Type Test
ERD	Error Rate of Decisions	QAR	Quick Access Recorder
EVD	EigenValue Decomposition	R/S	Range/Standard deviation
IF	Intrinsic Factors	S-R	Stimulus-Response
IQ	Intrinsic Qualities	SSE	Sum of the Squared Errors
I/O	Input/Output	TLD	TrLogDet
ITM	Information Theoretic Metric	VAP	Visual Attention Performance

## Introduction

### Backgrounds and motivations

Human error is a major source of air traffic control (ATC) accidents or incidents [1–6]. Luo [7] showed that 74.3% of accidents related to ATC of China are caused by air traffic controllers, or controllers for simplicity, instead of ATC equipments. The Aviation Safety Reporting System (ASRS) indicated that the aviation accidents are closely related with the *visual attention performance* (VAP) [8]. The objective of the controller is to resolve flight conflicts and ensure flight safety management subject to dealing with a large amount of visual and auditory information and making decisions *accurately*, *rapidly* and *stably* [9–11]. When there is hazardous weather, communication failures and/or aircraft mechanical failures, controllers will commit unsafe acts which lead to human errors, like “error/mistake”, “forgetting” and “omission”. It is necessary for controllers to concentrate and allocate attention so as to avoid flight accidents or incidents [12, 13]. The history of ATC shows that controllers’ misidentification may cause severe aviation accidents or incidents. On 11th October 2016, a serious runway incursion incident during takeoff occurred because of the controller’s erroneous operations in Shanghai Hongqiao International Airport of China [14, 15]. This incident was due to controller’s forgetting runway state and commanding mistake. It should be pointed out that there are two main factors for this incident: *lapse of attention* and *lack of expertise as a novice in ATC*.

A key issue for the cause of operational errors by novice controllers is the inadequate VAP [16]. According to the applied attention theory [17], visual misidentification may occur due to individual factors and attentional slips. Visual misidentification usually engenders errors in visual detecting and searching. There are quite a number of references about human perception processing [18–23], visual searching and cognition mechanism [24–27], which emphasize the mechanism of attention and play a fundamental role for human perception, cognition and action. There are two categories of factors influencing VAP: *extrinsic factors* (EF), which refer to the impersonal factors that affect attention or the factors that are not related to individual and affect attention, and *intrinsic factors* (IF), which refer to the personal-related factors that affect attention. Our emphasis is put on the IF influencing VAP and their systematic assessment. The inadequacy of VAP may cause serious human errors in civil aviation. The descriptions of the EF influencing VAP are abundant such as budget of search, complexity of task, noise or dark environment [21, 22]. However, the studies on the relation of human errors and the IF influencing VAP, such as attention span, attention stability, and distribution-shift of attention [20, 28–30], are deficient. Moreover, these IF are conceptual and there is no quantitative method to analyze them mathematically. It is necessary to analyze the VAP quantitatively by converting the conceptual IF into the computational counterparts, i.e., *intrinsic qualities* (IQ).

Intuitively and empirically, the controllers can be classified reasonably into four levels, i.e., excellent, good, moderate and unqualified. Theoretically, this classification can be implemented by clustering the features constructed from the IQ and evaluated with statistical inference. The determination of specific level which the control belongs to has a potential application in selecting air traffic controllers subject to detecting human errors and reducing the operation risk.

### Visual attention performances and clustering

It is feasible to analyze the VAP quantitatively with the help of our Neuron Type Test (NTT) system [31] and data collected (see S1 Appendix). Once the response sequences of ATC students are collected with NTT, we can define, compute and analyze the VAP by modeling the IF with mathematical methods. Technically, we take a three-step strategy to attain this objective:

1. **Modeling**: Based-on the response sequences, the feature space is constructed with measurable parameters proposed for IF with psychological experiments, mathematical and statistical theories.
2. **Clustering**: The ATC students characterized by the 2-dim or 3-dim features are classified via the *K*-means clustering algorithm automatically.
3. **Assessing**: Different clusters are evaluated by statistical inference in order to control the risk of human errors in aviation safety management by selecting potential professionals of ATC.

In civil aviation, the clustering method has been successfully applied in intelligent classification of air traffic controllers [32] and aeronautical information network intrusion detection [33]. On account of worldwide aircraft accidents between 1950s and 2010s, Christopher [34] and Olja [35] adopted the clustering method to predict the warning level of aircraft components and constructed an accident predictive models respectively. Normal and abnormal operations can be identified by trajectory clustering [36], which helps to surveillance the airspace efficiently. In 2012, main factors about approaching and the quick access recorder (QAR) data were analyzed by Zhou [37] with gray clustering method for assessing aircrew and improving flight safety. Vitali [38] studied the statistical regularities in ATM. In 2013, Monechi [39] analyzed the interrelation of safety data with flight trajectories and network metrics. In 2017, anomalous flight operations are identified from energy-based metrics and clustering [40] and Li et al. [41–43] studied the clustering and classification of IQ influencing the VAP of controllers.

With the purpose of controlling human errors and enhancing the safety performance in aviation operations, not only should we monitor the abnormal operations in time, but also assess the attention of operators efficaciously at the same time. The history of aviation safety shows that it is vital to pay more attention on operators instead of equipments [44]. In this paper, we propose systematic assessment on individual’s IF via the computational IQ by analyzing the response sequence with R/S analysis [31], *K*-means clustering algorithm [45] and statistic inference so as to identify the relation of human error and information processing level specified by the IF. We deem that the predictive quantification assessment for human errors caused by inadequacy of attention qualities could be used for selecting controller candidates and risk management.

### Roadmap, contents and organization

**Fig 1:**
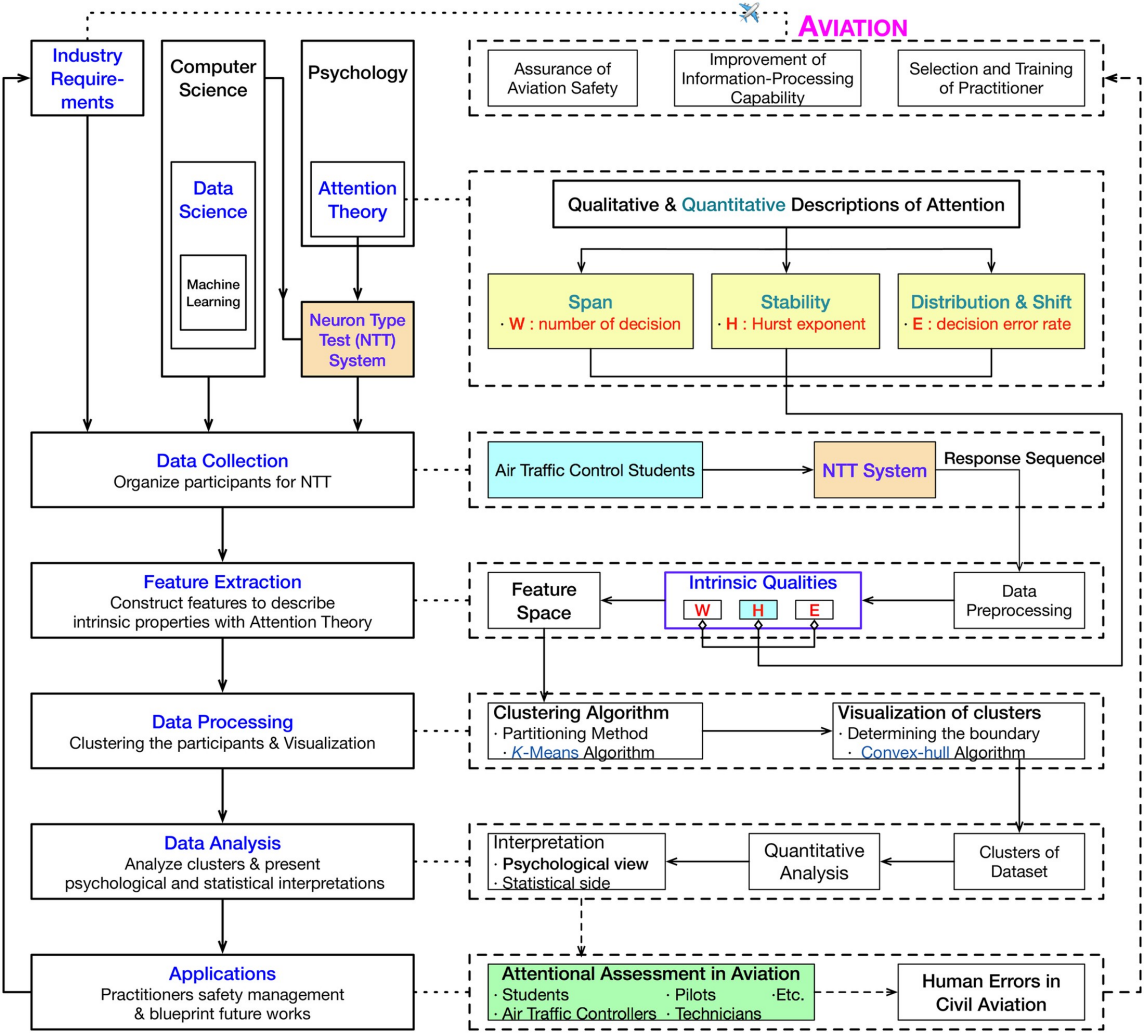
Aviation safety, visual attention theory and clustering algorithm: Logical hierarchy and technical implementation strategy.

For the purpose of quantifying the essential differences among controllers, a group of participants—air traffic control students—are invited to take two consecutive 12 minutes selective attention test with the “Ruleout-Ignore-Eliminate” rules. The experiments were carried with the help of the NTT system. The participants’ IF are coded in the response sequences collected in the experiment. Motivated by psychology and data science, we defined 2-dim and 3-dim feature spaces clustering the participants and assessing the IF with *K*-means clustering algorithm and statistical inference. The main ideas, logics and contents framework of this work are depicted in Fig 1.

The contents of this paper are organized as follows: the mathematical preliminaries are presented briefly in Section 2; the computational representation of IF in the sense of attention theory and data science are discussed in Section 3; the interpretation of the experiments about intrinsic factors with NTT are explained in Section 4; the intrinsic qualities are explained with the help of 2-dim and 3-dim clustering results in Section 5; finally the conclusions are given in Section 6. In the appendices, we give some introductions to the NTT system and *K*-means clustering algorithm.

## Mathematical preliminaries

### R/S analysis and Hurst exponent

In [31], the R/S analysis is used to analyze the Hurst exponent [46–48] of the reaction time data collected from the NTT system. For a discrete reaction time sequence ${\left\{X_{k}\right\}}_{k=0}^{L-1}$ with length *ℓ* = *d* × *n*, it can be segmented into *d* subsequences in which the *m*-th subsequence is ${\left\{X_{i}^{\left(m\right)}\right\}}_{i=0}^{n-1}$ where *m* ∈ {0, ⋯, *d* − 1} and

$$X_{i}^{\left(m\right)}=X_{mn+i},\;m\in \{0,\cdots ,d-1\}.$$ (1)

We can compute the local average *E*_*m*_, bias $Y_{i}^{\left(m\right)}$, cumulative deviate $Z_{i}^{\left(m\right)}$, range $R_{n}^{\left(m\right)}$ and standard deviation $S_{n}^{\left(m\right)}$ respectively as follows:

$$E_{m}=\frac{1}{n}\sum_{i=0}^{n-1}X_{i}^{\left(m\right)},\;m\in \{0,\cdots ,d-1\}$$ (2)

$$Y_{i}^{\left(m\right)}=X_{i}^{\left(m\right)}-E_{m},\;i\in \{0,\cdots ,n-1\}$$ (3)

$$Z_{i}^{\left(m\right)}=\sum_{j=0}^{i}Y_{j}^{\left(m\right)},\;i\in \{0,\cdots ,n-1\}$$ (4)

$$R_{n}^{\left(m\right)}=\max_{0\leq i\leq n-1}Z_{i}^{\left(m\right)}-\min_{0\leq i\leq n-1}Z_{i}^{\left(m\right)}$$ (5)

$$S_{n}^{\left(m\right)}=\sqrt{\frac{1}{n-1}\sum_{i=0}^{n-1}{\left[X_{i}^{\left(m\right)}-E_{m}\right]}^{2}}$$ (6)

Thus the average of the rescaled range for all subsequences of length *n* will be

$$\begin{array}{c} {\left(R/S\right)}_{n}=\frac{1}{d}\sum_{m=0}^{d-1}\frac{R_{n}^{\left(m\right)}}{S_{n}^{\left(m\right)}} \end{array}$$ (7)

In [47], Mandelbrot showed that the R/S statistics asymptotically follows the relation:

$$\begin{array}{c} {\left(R/S\right)}_{n}∼c\cdot n^{H}. \end{array}$$ (8)

Hence the Hurst exponent *H* can be estimated via least square approach according to (8). Usually, *H* ∈ (0, 1). For the reaction time sequences in NTT experiment, [31] shows that *H* > 0.5 means the process is persistent, *H* = 0.5 means that the process is near random, and *H* < 0.5 implies that the process is anti-persistent.

### Features and clusters

#### *n*-dim feature space and partition

The *n*-dim feature space is denoted by

$$\begin{array}{c} {\text{S}}^{n}=\{x={\left[x^{1},\cdots ,x^{n}\right]}^{⊺}:x^{i}\in R_{i}\subset R\} \end{array}$$ (9)

where the component *x*^*i*^ lies in the set *R*_*i*_ for each feature ***x***, and each *R*_*i*_ may be discrete or continuous. For any two features ***x*** and ***y*** in ${\text{S}}^{n}$, we use the Euclidean distance ∥***x*** − ***y***∥ to measure their difference although there are plenty of candidates of metric.

If the magnitudes of components for a feature point span a large scale, then we normalize each component in order to balance the contribution of each component. In this paper, we take the following min-max normalization step:

$$\begin{array}{c} x_{new}^{j}=\frac{x^{j}-x_{min}}{x_{max}^{j}-x_{min}^{j}},\;x_{new}^{j}\in \left[0,1\right],x^{j}\in R_{j} \end{array}$$ (10)

where

$$\begin{array}{c} x_{min}^{j}=\min_{x^{j}\in R_{j}}x^{j},\;x_{max}^{j}=\max_{x^{j}\in R_{j}}x^{j}. \end{array}$$ (11)

For simplicity, we will use the notation ***x*** for both the primitive feature ***x*** and its normalized version ***x***_*new*_. For the normalized feature space, we have *R*_*i*_ = [0, 1] and each feature is located in an *n*-dim unit cube

$$\begin{array}{c} {\text{C}}^{n}=\underset{n\;\text{terms}}{\underset{︸}{\left[0,1]\times \cdots \times [0,1\right]}}=\{x={\left[x^{1},\cdots ,x^{n}\right]}^{⊺}:0\leq x^{i}\leq 1\}. \end{array}$$ (12)

For the index set {1, ⋯, *N*}, it can be partioned into *K* disjoint subsets Φ_1_, ⋯, Φ_*K*_ such that $\left\{1,2,\cdots ,N\right\}=\cup_{k=1}^{K}\Phi_{k}$ and Φ_*j*_ ∩ Φ_*i*_ = ∅ for *i* ≠ *j*. Let |*A*| be the cardinality of set *A* and |Φ_*k*_| = *N*_*k*_, then we have $|\cup_{k=1}^{K}\Phi_{k}|=N$. Similarly, for the data set

$$\begin{array}{c} S^{n}=\{x_{k}={\left[x_{k}^{1},\cdots ,x_{k}^{j},\cdots ,x_{k}^{n}\right]}^{⊺}:k\in \{1,\cdots ,N\}\}={\left\{x_{k}\right\}}_{k=1}^{N}, \end{array}$$ (13)

which consists of *N* discrete *n*-dim features, we can divided it into *K* disjoint subsets $S_{1}^{n},\cdots ,S_{K}^{n}$ as follows

$$\begin{array}{c} S_{k}^{n}=\{x_{i_{k}}={\left[x_{i_{k}}^{1},\cdots ,x_{i_{k}}^{n}\right]}^{⊺}\in S^{n}:i_{k}\in \Phi_{k}\},\;k\in \{1,\cdots ,K\}. \end{array}$$ (14)

In consequence,

$$\begin{array}{c} S^{n}=\overset{K}{\underset{k=1}{\cup }}S_{k}^{n},\;S_{j}^{n}\cap S_{k}^{n}=\emptyset \;\text{for}\;j\neq k. \end{array}$$ (15)

and $|S|=\sum_{k=1}^{K}|S_{k}^{n}|=N$. For the purpose of clustering, we use the disjoint sets $S_{1}^{n},\cdots ,S_{K}^{n}$ to denote the *K* different clusters.

For each feature point ***x***_*j*_ in $S^{n}=\underset{k}{\cup }S_{k}^{n}$, we use the binary indicator variable *I*_*kj*_, known as the 1-of-*K* coding scheme [49],

$$\begin{array}{c} I_{jk}=I_{S_{k}^{n}}\left(x_{j}\right)=\{\begin{array}{c} 1,\;x_{j}\in S_{k}^{n};\\ 0,\;x_{j}\notin S_{k}^{n} \end{array} \end{array}$$ (16)

to label how to assign the point ***x***_*j*_ into the cluster of interest. If ***x***_*j*_ is assigned to the *k*-th cluster $S_{k}^{n}$, i.e., $x_{j}\in S_{k}^{n}$, then *I*_*jk*_ = 1, otherwise *I*_*jk*_ = 0.

#### Statistics and metrics for clusters

The centroid of a given cluster $S_{k}^{n}$ is the average of the features in the cluster $S_{k}^{n}$, i.e.,

$$\begin{array}{c} \mu_{k}=\mu \left(S_{k}^{n}\right)=\frac{1}{\left|S_{k}^{n}\right|}\sum_{x\in S_{k}^{n}}x. \end{array}$$ (17)

For the features ***x***_*i*_ and ***x***_*j*_ in feature space *S*^*n*^, their dissimilarity is measured by their Euclidean distance. viz.,

$$\begin{array}{c} {\mathrm{dist}}_{E}\left(x_{i},x_{j}\right)=∥x_{i}-x_{j}∥ \end{array}$$ (18)

For clusters $S_{k}^{n}$ and $S_{i}^{n}$, we use the Euclidean distance of their corresponding centroids to represent their distance

$$\begin{array}{c} {\mathrm{dist}}_{E}\left(S_{k}^{n},S_{i}^{n}\right)=∥\mu_{k}-\mu_{i}∥,\;\forall i,k\in \{1,\cdots ,N\}. \end{array}$$ (19)

Without doubt, the distance of clusters is an inter-class metric which describes the degree of separation of two clusters. For each cluster $S_{k}^{n}$, the standard deviation of features is defined by

$$\begin{array}{c} \sigma_{k}=\sigma \left(S_{k}^{n}\right)=\sqrt{\frac{1}{|S_{k}^{n}|-1}\sum_{x\in S_{k}^{n}}{∥x-\mu_{k}∥}^{2}}. \end{array}$$ (20)

Note that we use the unbiased estimation of variation since *N*_*k*_ = |Φ_*k*_| may not be efficiently large. Intuitively, the smaller the standard deviation is, the better the cluster is. The standard deviation per dimension is defined by

$$\begin{array}{c} {\bar{\sigma }}_{k}=\frac{\sigma (S_{k}^{n})}{n}, \end{array}$$ (21)

which can be used to compare the clusters with different dimensions when the impact of the dimension of features is considered in the practical problems.

Although the standard deviation is simple and useful, it is difficult to describe the orientation of the features in a cluster. It is necessary for us to consider the (unbiased) estimation of the covariation matrix of $S_{k}^{n}$, i.e.,

$$\begin{array}{c} \Sigma_{k}^{n}=\text{Cov}\left(x_{i}I_{S_{k}^{n}},x_{j}I_{S_{k}^{n}}\right)=\frac{1}{|S_{k}^{n}|-1}\sum_{x\in S_{k}^{n}}\left(x-\mu_{k}\right){\left(x-\mu_{k}\right)}^{⊺}. \end{array}$$ (22)

For the features ***x***_*i*_ and ***x***_*j*_ in *S*^*n*^, their correlation coefficient is defined by

$$\begin{array}{c} \mathrm{Cr}\left(x_{i}I_{S_{k}^{n}},x_{j}I_{S_{k}^{n}}\right)=\frac{\text{Cov}(x_{i}I_{S_{k}^{n}},x_{j}I_{S_{k}^{n}})}{\sqrt{\text{Cov}(x_{i}I_{S_{k}^{n}},x_{i}I_{S_{k}^{n}})}\cdot \sqrt{\text{Cov}(x_{j}I_{S_{k}^{n}},x_{j}I_{S_{k}^{n}})}}. \end{array}$$ (23)

For the purpose of comparing the difference of clusters, we define the *TrLogDet* (TLD) divergence for the clusters $S_{k}^{n}$ and $S_{i}^{n}$ by

$$\begin{array}{c} {\text{div}}_{T}\left(S_{k}^{n},S_{i}^{n}\right)=\frac{1}{n}[\mathrm{tr}\left(\Sigma_{k}\Sigma_{i}^{-1}\right)-\log\det\left(\Sigma_{k}\Sigma_{i}^{-1}\right)-n]. \end{array}$$ (24)

Then we introduce the *TrLogDet* (TLD) distance

$$\begin{array}{c} {\mathrm{dist}}_{T}\left(S_{k}^{n},S_{i}^{n}\right)=\frac{1}{2}[{\text{div}}_{T}\left(S_{k}^{n},S_{i}^{n}\right)+{\text{div}}_{T}\left(S_{i}^{n},S_{k}^{n}\right)]. \end{array}$$ (25)

Note that the expression for TLD in (24) is different from LogDet in [50, 51] since the factor 1/*n* is introduced for the purpose of eliminating the impact of the dimension of the data and the trace operation is emphasized here. It is easy to show that the TrLogDet distance satisfies the axioms of distance:

- (non-negative) ${\mathrm{dist}}_{T}\left(S_{k}^{n},S_{i}^{n}\right)\geq 0$ and ${\mathrm{dist}}_{T}\left(S_{k}^{n},S_{k}^{n}\right)=0$;
- (symmetric) ${\mathrm{dist}}_{T}\left(S_{k}^{n},S_{i}^{n}\right)={\mathrm{dist}}_{T}\left(S_{i}^{n},S_{k}^{n}\right)$;
- (triangle inequality) ${\mathrm{dist}}_{T}\left(S_{k}^{n},S_{i}^{n}\right)+{\mathrm{dist}}_{T}\left(S_{i}^{n},S_{j}^{n}\right)\geq {\mathrm{dist}}_{T}\left(S_{k}^{n},S_{j}^{n}\right)$.

The TLD distance measures the dissimilarity of two clusters and it is a metric of inter-clusters.

For the clusters $S_{k}^{n}$ and $S_{i}^{n}$, when $\Sigma_{i}=\text{Cov}\left(S_{i}^{n}\right)=I$ is the identity matrix, namely an “ideal” or “standard” cluster, we immediately have

$$\begin{array}{c} \rho_{k}=\rho \left(S_{k}^{n}\right)=\frac{1}{n}[\mathrm{tr}\left(\Sigma_{k}\right)-\log\det\left(\Sigma_{k}\right)-n]=-1+\frac{1}{n}\sum_{j=1}^{n}\left(\lambda_{k}^{j}-\log\lambda_{k}^{j}\right). \end{array}$$ (26)

In this case, $\rho (S_{k}^{n})$ is the so called *Information-Theoretic Metric* (ITM) [50, 51] per dimension, which is a positive, strictly convex and spectral function. Particularly, if **Σ**_*k*_ = ***I*** = diag(1, ⋯, 1), then *ρ*_*k*_ = 0. Usually, the ITM *ρ*_*k*_ can be used as a divergence metric for the cluster $S_{k}^{n}$, just as the standard deviation *σ*_*k*_ does.

The choice of *K* in the *K*-means algorithm is a key issue in clustering. The value of *K* has direct impact on the clustering results, which is related to the *sum of the squared errors* (SSE) for *K* clusters:

$$\begin{array}{c} \mathrm{SSE}=\sum_{k=1}^{K}\mathrm{SSE}\left(S_{k}^{n}\right)=\sum_{k=1}^{K}\sum_{x\in S_{k}^{n}}{∥x-\mu_{k}∥}^{2} \end{array}$$ (27)

where $\mathrm{SSE}(S_{k}^{n})$ is the sum of squared errors for the *k*-th cluster $S_{k}^{n}$.

## Modeling the intrinsic factors influencing visual attention performances

### Conceptions

Logically, there are three key issues to be done for quantitatively assessing the IF influencing VAP:

1. proposing an abstract description for IF by mathematical modeling;
2. developing an interactive system to collect data which preserve the intrinsic and invariant properties interested naturally;
3. finding proper ways to analyze the data collected and give reliable, effective and practical assessment methods for the IF.

Conceptually, a reasonable data collecting system for analyzing the IF should have the following elements:

- Structure: the stimulation paradigm should be an Stimulus-Response (S-R) model, which can be represented by an Input/Output (I/O) model theoretically;
  - Input: non-semantic symbols serve as visual stimulus;
  - Output: the response of participant is the decision which includes reaction time and the symbolized description of correctness;
- Function: the data collected with the system can be used to analyze the attention span, stability of attention, distribution-shift of attention for individuals;
- Interface: the human-machine interaction interface should be friendly and easy to be used for the users.

According to these elements, the NTT system has been well developed [31] and used to collect the response sequence of individuals, in which the *i*-th response ${\langle y_{i},\tau_{i}\rangle }_{p}=\langle y_{i}^{p},\tau_{i}^{p}\rangle$ for the *p*-th individual consists of two components:

- symbolic decision, denoted by *y*_*i*_ such that *y*_*i*_ ∈ {*Y*, *N*, *I*} where *Y*, *N* and *I* stand for “Yes”, “No” and “Ignore” respectively;
- reaction time, denoted by *τ*_*i*_ such that *τ*_*i*_ > 0.

The length of the response sequence refers to the count of responses in a time interval specified by the experimental setup. Usually, the length is different for different individuals and it can be regarded as a random variable mathematically.

### Modeling the intrinsic factors: Qualitative and quantitative descriptions

The performance of attention is affected by a variety of factors, which can be divided into two fundamental categories [29, 30]: extrinsic factors and intrinsic factors. Extrinsic factors include mission objectives, plans, operating procedures and so on. Alternatively, the intrinsic factors include knowledge, skills, experience, personal attention level and so on.

In terms of human errors in aviation, the *rapidness*, *accuracy*, and *stability* of the behaviors of operators are essential for avoiding incidents or accidents in case of emergencies. There are two famous cases in civil aviation in the recent decade: the forced landing of New York National Airline on Hudson River on 15 January 2009 (engine failed) [52] and the abdomen landing of Polish Airline (landing gear failed to lay down) on 1 November 2011. These three characteristics are closely related to the IF influencing VAP for aircraft operators.

Currently, there is a lack of a complete test approach for attention performances, thus we can only assess some aspects of human behaviors characterized by some components of attention performances [53]. In the sense of psychology, the interests of the assessment of IF in this work consist of three aspects: attention span, attention stability, distribution-shift of attention. With the response sequence collected by the NTT system, it is feasible to measure and assess the IF with observable data.

**Fig 2:**
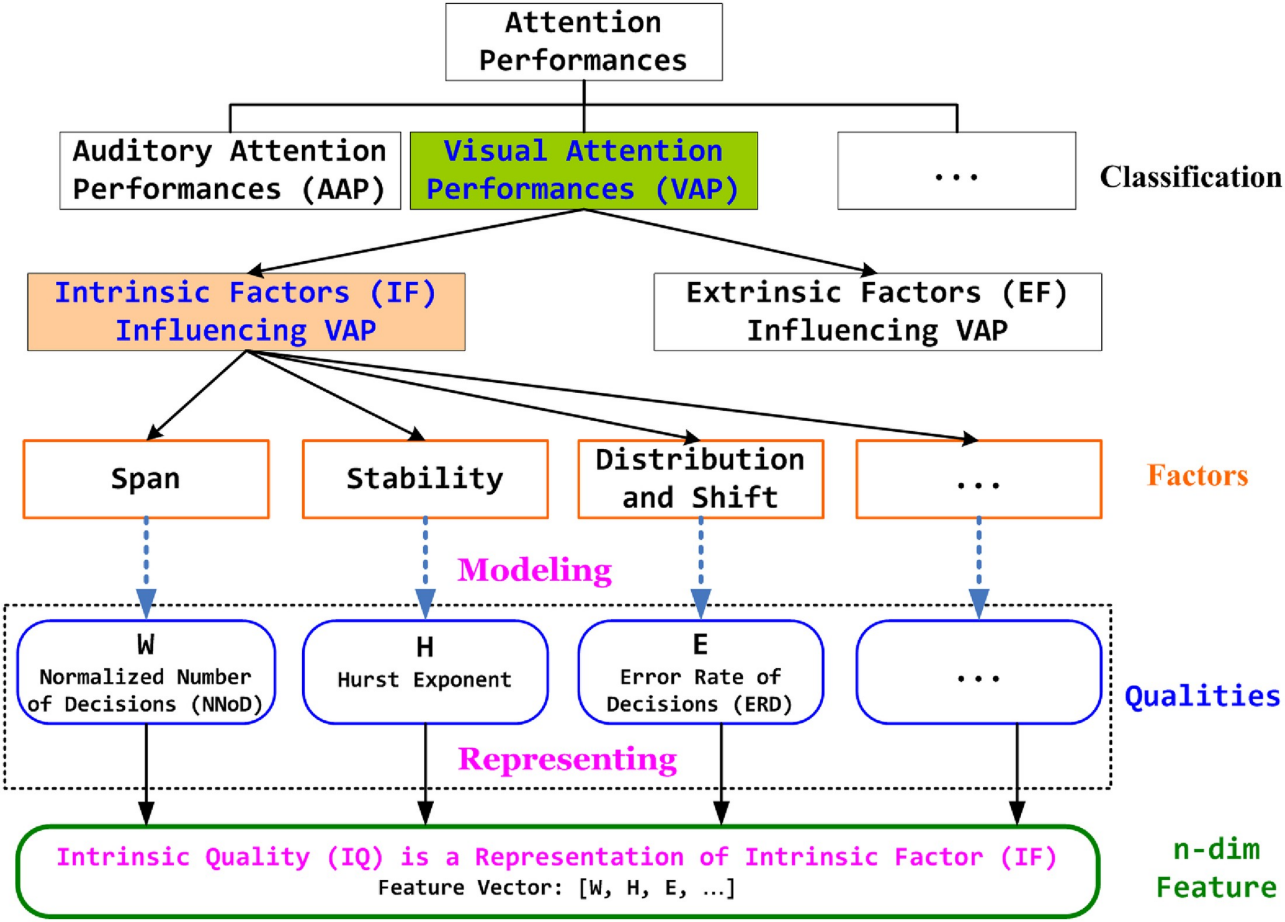
Visual attention performances: From concepts to feature vectors.

For the convience of understanding the ideas and methods for modeling the IF influencing VAP and the corresponding IQ of interest, we figure out a block diagram for VAP, see Fig 2.

#### Attention span—Number of decisions and its normalization

Attention span is the amount of concentrated time one can spend on a task without becoming distracted. If the concentrated time interval is fixed, then attention span refers to the amount of perceived or recognized objects during a given period for an individual. Intuitively, the more decisions the participant makes in each test via NTT System, the faster the participant responses in a given time interval (2 × 12 = 24 minutes). Therefore, the *number of decisions* (NoD) made in the fixed time can be used as a data feature to describe the operational capacity of a single participant. Attention span relates to the individual’s information processing intensity, which determines the number of the objects perceived. NoD maps the number of percepts and furthermore reflect the degree of rapidness which serves as the assessment index for attention span.

Mathematically, the NoD is denoted by *I* and its value must be a positive integer, i.e., I∈N. In our NTT test, we have *I* ∈ [200, 800] according to the setup of the practical experiments of NTT. For the sake of data processing and pre-conditioning in data science, we take the following *normalized number of decisions* (NNoD) with min-max normalization step according to (10) and (11)

$$\begin{array}{c} W=\frac{I-I_{min}}{I_{max}-I_{min}}\in \left[0,1\right]. \end{array}$$ (28)

#### Attention stability—Hurst exponent

Stable attention relates to the goal of the activity, the level of understanding of the task, the interest on the object, the degree of motivation to engage in the activity, emotional state, activeness of thought and healthy state. Attention stability describes the individual’s quality of keeping attention on a certain object or an activity for a long duration [54]. In civil aviation, the attention stability reflects ATM controllers’ ability for tracking aircrafts among sectors and/or flights en rout continuously and smoothly.

Based on the authors’ previous work [31], the Hurst exponent [55], denoted by *H*, is a good feature for the reaction time data such that the latent information and long term correlation can be characterized efficiently. *The Hurst exponent H is a natural and intrinsic parameter for representing the level of attention stability.* In a heuristic way, there are two aspects which should be noted:

- The available methods for describing attention stability are based on average, range of reaction time and the standard deviation independently [56]. On the contrary, the Hurst exponent *H* integrates all of these factors into a single and simple statistical invariant in the sense of affine transformation by its definition.
- This statement is a fundamental hypothesis and its effectiveness should be verified and validated by practical cases and possible applications. In the sense of mathematical modeling of IF, it is an abstraction for describing the attention stability with Hurst exponent *H*. For our response sequences obtained by NTT system based on Stimulus-Response model, this hypothesis is acceptable and the abstraction leads to interesting and reasonable results when clustering with experimental data.

#### Distribution-shift of attention—Error rate of decisions

Distribution of attention indicates distributing attention on distinct objects simultaneously, which reflects the capability of processing multiple tasks parallelly. Shift of attention refers to transferring the attention intentionally from one object, or an activity, or an operation, to another.

“Error” is usually a conscious behavior of individual. It is due to the lack of practitioners’ ability, behavioral fault, unfamiliarity with the rules of operation, the lack of relevant knowledge and other factors caused by operational errors or fault. The job of air traffic controller is to monitor, control and service the flight activities of aircrafts by using communication, navigation, monitoring techniques and methods to prevent aircraft collision and guarantee the safety, which covers the different stages of perception of information, analysis, decision-making and implementation. During this information processing, the controller’s memory and attention play the dominate roles in the accuracy of his operation. *Error*, *omission*, and *forgetting*—three typical forms of human mistakes—are caused by the lapse of distribution-shift of attention. We use the *error rate of decisions* (ERD), denoted by *E*, to represent the correctness of decisions which indicates the distribution-shift of attention. In the sense of aviation operation, ERD is of significance for the air traffic controllers. For the tests with NTT System, it is defined by

$$\begin{array}{c} E=\frac{\text{Number}\;\text{of}\;\text{Incorrect}\;\text{Decisions}}{\text{Number}\;\text{of}\;\text{Decisions}}\times 100\%\in \left[0,1\right]. \end{array}$$ (29)

### Representation of IF and feature space

The representation of IF is IQ, which leads to the feature space with high dimensional features generally, please see Fig 2.

#### 2-dim feature space

In practical problems, some features of data may not be easily acquired. Hence, the analysis for low dimensional sample space may be indispensable. On the basis of the modeling of IF, we have obtained three features and constructed interested 2-dim and 3-dim feature spaces.

For the 2-dim feature space, we use the Hurst exponent *H* and the NNoD *W* to construct the feature space which consists of points [*W*, *H*]^⊺^ such that *H* ∈ [0, 1] according to [31] and *W* ∈ [0, 1] by normalization. We should remark it is not a wise choice if we use the NoD *I* instead of the NNoD *W*. Actually, the order of magnitudes of *H* and *I* are 10^−1^ and 10^2^ respectively, and the weight for the two components of each feature vector [*I*, *H*]^⊺^ are the same. In consequence, the Euclidean distance of the feature vectors is dominated by the NoD *I*. To avoid such a result, it is necessary to use the normalized version *W* rather than the primitive *I* as the suggestion proposed in [57].

Consequently, the *p*-th feature vector $x_{p}^{2}={\left[H_{p},W_{p}\right]}^{⊺}$, which corresponds to the *p*-th individual/participant and is constructed by Hurst exponent and NNoD, are located in the normalized feature space ${\text{C}}^{2}$ (a unit square), i.e.,

$$\begin{array}{c} x_{p}^{2}\in {\text{C}}^{2}=\{{\left[W,H\right]}^{⊺}:0\leq W\leq 1,0\leq H\leq 1\}. \end{array}$$

For the *p*-th participant and the corresponding response sequence ${\langle y_{i}^{p},\tau_{i}^{p}\rangle }_{i\in N}$, we can find that:

- *I*_*p*_ is the number of decisions determined by the reaction time sequence ${\left\{\tau_{i}^{p}\right\}}_{i\in N}$;
- *W*_*p*_ can be calculated from *I*_*p*_ with the help of Eqs (10) and (11);
- *H*_*p*_ can be computed from the reaction time sequence ${\left\{\tau_{i}^{p}\right\}}_{i\in N}$.

#### 3-dim feature space

Meanwhile, it is not sufficient if only 2-dim clustering is taken for analyzing our problem. For the sake of more deep understanding of the problem, we also constructed 3-dim feature space by adding the component *E*, i.e., the ERD.

Hence, the *p*-th 2-dim feature $x_{f}^{2}$ now turns into a 3-dim vector $x_{p}^{3}={\left[W_{p},H_{p},E_{p}\right]}^{⊺}$, which is constructed by NNoD *W*, Hurst exponent *H* and ERD *E*. Obviously, each $x_{p}^{3}$ is located in the 3-dim normalized feature space ${\text{C}}^{3}$ (a unit cube), i.e.,

$$\begin{array}{c} x_{p}^{3}\in {\text{C}}^{3}=\{{\left[W,H,E\right]}^{⊺}:0\leq W\leq 1,0\leq H\leq 1,0\leq E\leq 1\}. \end{array}$$

For the the *p*-th participant and its decision sequence ${\left\{y_{i}^{p}\right\}}_{i\in N}$, the ERD *E*_*p*_ can be determined easily.

#### Eigen features, measurability and operation risk

Mathematically, the selection of the basis is not unique for the *n*-dim feature space ${\text{C}}^{n}$. However, different bases are equivalently and they can be related with an invertible transition matrix. For the general features, it is sufficient for us if we take any form of the basis. However, if the clusters are considered, we need the concept of eigen-feature and eigen space.

For the *k*-th covariance matrix $\Sigma_{k}^{n}$ of cluster $S_{k}^{n}$ defined by (22) in ${\text{C}}^{n}$, we denote its eigenvalue decomposition (EVD) as

$$\begin{array}{c} \Sigma_{k}^{n}=\sum_{i=1}^{n}\lambda_{i}^{\left(n,k\right)}e_{i}^{\left(n,k\right)}{\left(e_{i}^{\left(n,k\right)}\right)}^{⊺},\;1\leq k\leq K \end{array}$$ (30)

where $\lambda_{1}^{\left(n,k\right)},\lambda_{2}^{\left(n,k\right)},\;\cdots \;,\lambda_{n}^{\left(n,k\right)}\in R$ are the eigenvalues and $e_{1}^{\left(n,k\right)},e_{2}^{\left(n,k\right)},\;\cdots \;,e_{n}^{\left(n,k\right)}\in R^{n\times 1}$ are the *eigen-features* of the cluster $S_{k}^{n}$. Usually, the eigenvalues are sorted with decreasing order, i.e., $\lambda_{1}^{\left(n,k\right)}\geq \lambda_{2}^{\left(n,k\right)}\geq \cdots \geq \lambda_{n}^{\left(n,k\right)}$. The subspace spanned by the eigen-feature $e_{i}^{\left(n,k\right)}$ is called the *i*-th eigen space if the eigenvalue $\lambda_{i}^{\left(n,k\right)}$ is not degenerate. For simplicity, we take the following notation

$$\begin{array}{c} \text{Eig}\left(\Sigma_{k}^{n}\right)=\{\lambda_{1}^{\left(n,k\right)},\lambda_{2}^{\left(n,k\right)},\cdots ,\lambda_{n}^{\left(n,k\right)}\}. \end{array}$$ (31)

It should be noted that all of the eigenvalues are positive since the covariance matrix is positive.

If $e_{i}^{\left(n,k\right)}={\left[0,0,\cdots ,1,\cdots ,0\right]}^{⊺}$ where only the *i*-th position is 1, then the eigen-feature is standard. This fact is very important for our problem in which *W*, *H* and *E* are three components of the features concerned. In the sense of attention theory in psychology, the IF or equivalently IQ are not coupled and the *i*-th component of the IQ feature is *directly measurable* since *W*, *H* and *E* can be computed directly with the data collected in the NTT experiments. Generally, the eigen-feature $e_{i}^{\left(n,k\right)}$ is not standard and there are more than one factors/components should be considered simultaneously. On the other hand, this implies that each cluster has its own eigen-features and manifests its characteristics, thus the corresponding operators of civil aviation may have specific operation risk. Logically, we have the chain “cluster—eigen-features—operation-risk” for the aviation safety about air traffic control.

### Levels of intrinsic qualities

Here we define four levels—*High, Moderate, Low and Bottom*—which are sequencing the intrinsic visual attention quality from “great” to “trivial”. We now give some remarks for the levels of IQ:

- The bottom *W* indicates that the participants’ reaction is very slow and it costs much time for making a decision. Experimentally, the process for making symbolic decision *y*_*i*_ ∈ {*Y*, *N*, *I*} is long and the speed is very slow in the sense of statistics. Psychologically, it implies that the attention span of the participants is small. On the contrary, high *W* means fast reaction and large attention span.
- The moderate *H*, say *H* ∼ 0.6, shows that there is a weak positive correlation in the sense of long term time [31]. Therefore, the attention stability of the participants is considerable.

## Testing experiments and implementation of clustering algorithm

### Setup of experiments

We seriously declare that the Ethics Committee of Research Institute of Civil Aviation Safety approved this research and the consent procedure. Given that the present study only measures simple behavior responses, it does not elicit adverse physiological and psychological reactions from the participants. Thus, the institutional ethics committee waived the need for written informed consent from the participants. After all procedures had been explained by one of the researchers, those participants who decided to join the study could get an invitation code to access the program, which was the representation of their oral consent. Participants have the right to withdraw from the study at any time, and the researcher counted four participants who quited from the study in total. The participants are college students and they are required to read some simple symbols on the computer screen and make some harmless decisions by clicking the mouse [31].

In this work, the 168 participants involved are undergraduate and graduate students (20 ∼ 25 years old) selected from the College of Air Traffic Management in Civil Aviation University of China. In other words, the data collected for the systematic assessment of IF are based on air traffic control students. It should be pointed that the testing conditions of experiments are considered seriously:

- The participants involved in the NTT test are in a good mental state, i.e., with no sickness or being lack of sleep in the past 48 hours.
- The test environment is quiet and isolated from any observable natural and/or artificial interruptions.

In each NTT experiment, each participant is required to finish 2 successive testing tasks [31] within 2 × 12 minutes. In each task, each participant is guided to read lots of positive/negative non-semantic symbols and is asked to make decisions simultaneously. Without doubt, different participant will have different response sequence, and also different NoD in the same time interval due to the different reaction time in each decision operation. After data cleaning, the responses of 143 participants are kept and 25 records are abandoned. For the final data set, please see S1 Appendix.

### Constructing features for intrinsic qualities

The selected features and the number of features will affect the clustering result. For a prototype modeling, the data feature extraction shall be described firstly. After the pre-processing of data, the features of interested are extracted from the reaction time sequence of each participant. As a companion of Fig 2, the pseudo-code for the feature extraction for particilants in NTT is given in Algorithm 1.

**Algorithm 1:** *n*-dim Features Extraction for Participants

**Input:** Dimension *n* ∈ {2, 3}, response sequences ${\langle y_{i}^{p},\tau_{i}^{p}\rangle }_{i\in N}$ of *N* participants.

**Output:** Feature vectors ${\left\{x_{p}^{n}\right\}}_{p=1}^{N}$, where $x_{p}^{n}={\left[x_{1,p}^{n},\cdots ,x_{i,p}^{n},\cdots ,x_{n,p}^{n}\right]}^{⊺}$ of IQ.

1: Preconditioning of the input response sequences.

2: **for** *p* ∈ {1, ⋯, *N*} **do**

3:  Compute $W_{p}^{n}$ by Eq (28) with the decision sequence ${\left\{y_{i}^{p}\right\}}_{i\in N}$.

4:  Set the first component of $x_{p}^{n}:\;x_{2,p}^{n}=W_{p}^{n}$

5:  Compute $H_{p}^{n}$ by Eqs (1)∼(8) with the reaction time sequence ${\left\{\tau_{i}^{p}\right\}}_{i\in N}$.

6:  Set the second component of $x_{p}^{n}:\;x_{1,p}^{n}=H_{p}^{n}$.

7:  **if** *n* == 3 **then**

8:   Calculate $E_{p}^{n}$ by Eq (29) with the decision sequence ${\left\{y_{i}^{p}\right\}}_{i\in N}$.

9:   Set the third component of $x_{p}^{n}:\;x_{3,p}^{n}=E_{p}^{n}$

10:  **end if**

11: **end for**

12: **return** Feature vectors ${\left\{x_{p}^{n}\right\}}_{p=1}^{N}$.

### Implementation of *K*-means clustering algorithm

#### Applicability of *K*-means clustering algorithm

The advantage of *K*-means lies in its simple interpretation due to the intuitive and reliable clustering results. However, there are also some disadvantages for this algorithm: the possibility of convergence to local optimal and high computational complexity for large scale sample spaces. In our experiment, the data set collected from the NTT system is a small scale, thus the *K*-means algorithm works well.

#### Choice of *K*

For the implementation of *K*-means clustering algorithm, the *K* value can not be too large. The cross-validation method is used to select the *K* value. The majority of cases of selection are relying on the prior belief or intrinsic characteristics of the data. According to the characteristics of the NTT data set, the data set is divided into four categories which aims at classifying all of the participants into four levels: excellent, good, moderate and unqualified. The objective of clustering the feature points corresponding to the controllers (or air traffic control students), includes three folds:

- exploring the potential regulation for classifying controllers into different levels;
- predicting the lapse of attention in the practical operations in air traffic control by quantitative analysis;
- improving the IQ of controllers via professional training.

In our recent studies [41–43], the characteristics of attention quality of controllers are described and the differences between controllers and ATC students are revealed according to the types of attention quality. The results obtained show that:

- both controllers and ATC students could be classified into four types according to attention quality;
- the qualified controllers concerned show large attention span, strong attention stability, rational attention switching and improper attention distribution;
- there is a significant difference between full time controllers and ATC students.

#### Pseudocode for clustering algorithm

For the details of *K*-means algorithm, please refers to [45]. The 2-dim and 3-dim feature spaces of interest have been obtained from the feature extraction, and serve as the input of the *K*-means algorithm. Mathematically, we have $x_{j}=x_{k}^{2}$ or $x_{j}=x_{k}^{3}$ for the *j*-th feature when clustering.

### Clustering with experimental data from ATC students

**Fig 3:**
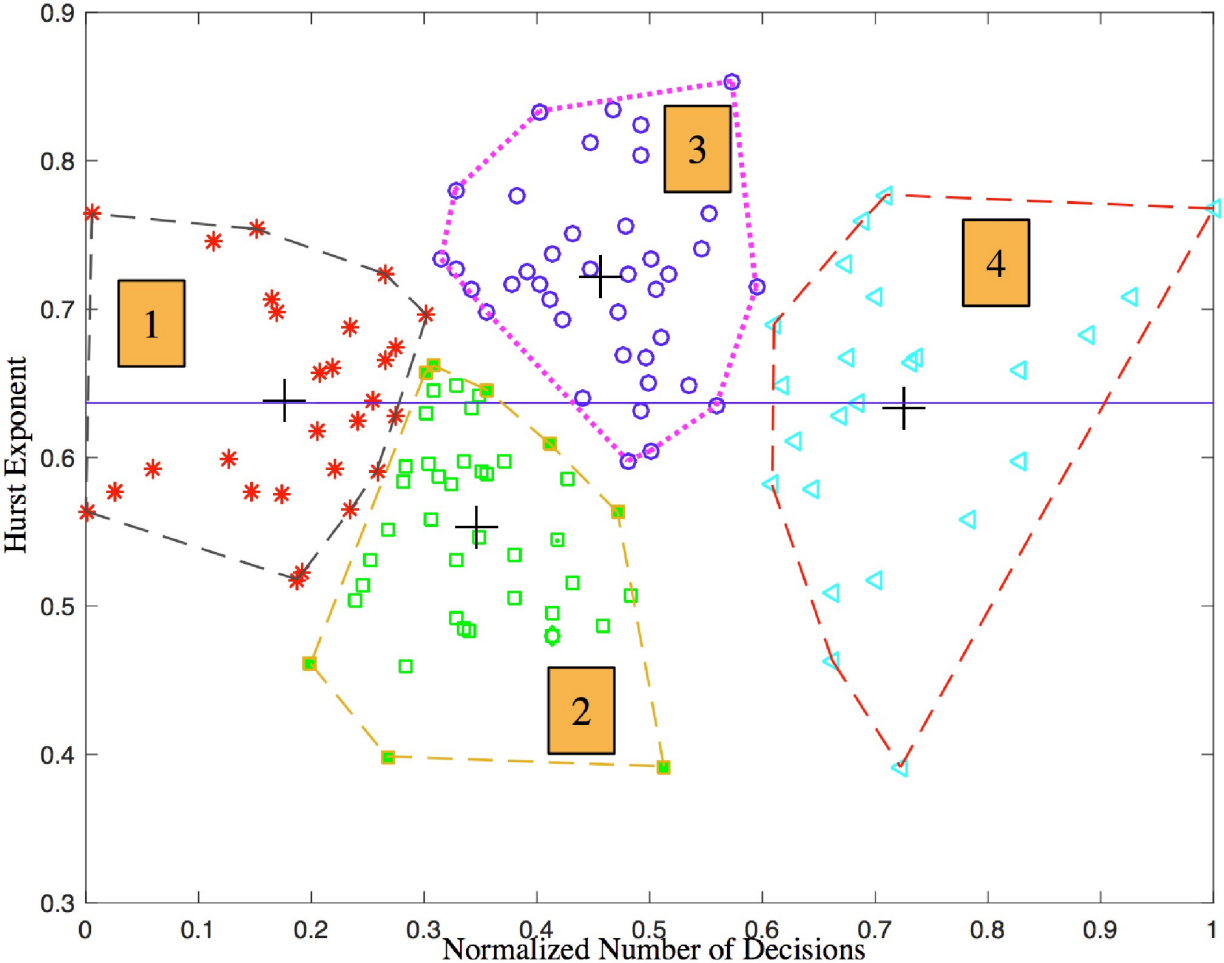
Clusters of 2-dim features [*W*, *H*]^⊺^ with boundaries generated by convex hull algorithm.

**Fig 4:**
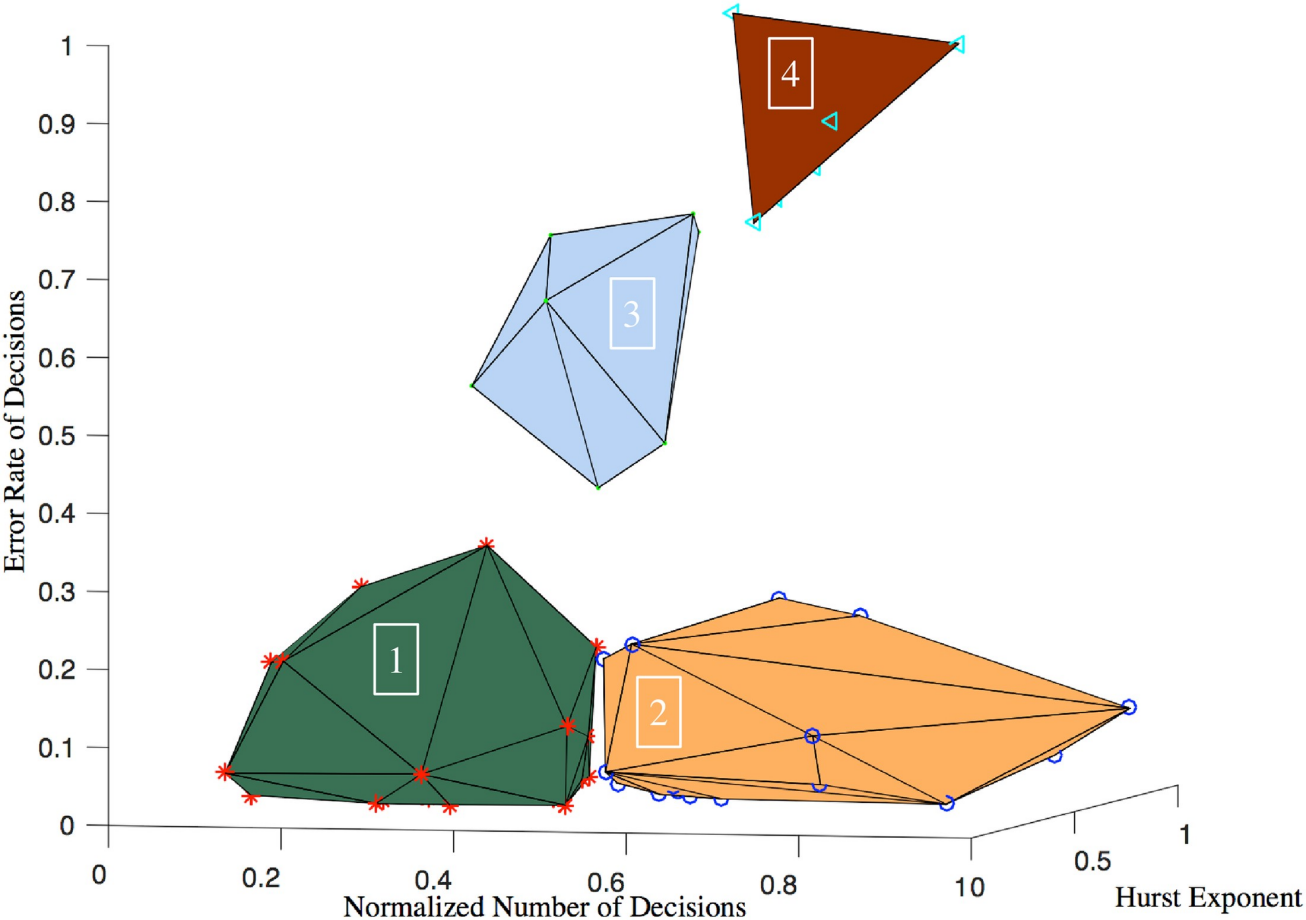
Clusters of 3-dim features [*W*, *H*, *E*]^⊺^ with contours.

The data obtained from the NTT System are analyzed carefully and systematically. The distribution of clusters can be visualized intuitively with 2-dim and 3-dim clustering. It is necessary for us to explain why both 2-dim and 3-dim clustering are valuable for classifying air traffic control problem. The key issue lies in the level of information processing of ATC students in the sense of psychology:

- the 2-dim clustering analysis with *K*-means algorithm is mainly based on the Hurst exponent and the workload, which reflects the speed and stability of information processing of the ATC students (see Fig 3);
- the 3-dim clustering analysis also reflects the accuracy of information processing of the ATC students (see Fig 4) if compared with the 2-dim clustering analysis;
- in general, the 3-dim clustering analysis is more comprehensive than the 2-dim clustering analysis for describing and explaining the level of information processing of the ATC students.

#### 2-dim clustering

The 2-dim feature space is taken for the 2-dim clustering. Eq (14) shows that the set of 2-dim feature vectors

$$\begin{array}{c} S^{2}=\{x_{i}\in {\text{C}}^{2}:1\leq i\leq N\} \end{array}$$ (32)

can be partioned into *K* = 4 disjoint subsets $S_{1}^{2},S_{2}^{2},S_{3}^{2}$ and $S_{4}^{2}$, where

$$\begin{array}{c} S_{k}^{2}=\{x_{i_{k}}={\left[W_{i_{k}},H_{i_{k}}\right]}^{⊺}:i_{k}\in \Phi_{k}\},\;1\leq k\leq K. \end{array}$$

With the purpose of partitioning different clusters with a distinct visualization, here we use Graham’s Scan [58] with a quasi-linear time computational complexity to find the convex hull of the feature points in our problem, i.e. the contours of clusters. This procedure simplifies the step of separating different clusters and avoid the complex methods for determining the border of clusters in clustering algorithms. Intuitively, Fig 3 shows that the third cluster is the best when the 2-dim clustering is taken because of the high stability of attention (large *H*) and good attention span (acceptable *W*).

#### 3-dim clustering

The 3-dim feature space is taken for 3-dim clustering. Eq (14) shows that the set of 3-dim discrete feature vectors

$$\begin{array}{c} S^{3}={\left\{x_{i}^{3}\right\}}_{i=1}^{N}\subset {\text{C}}^{3} \end{array}$$ (33)

can be partitioned into *K* = 4 disjoint subsets $S_{1}^{3},S_{2}^{3},S_{3}^{3},S_{4}^{3}$ where

$$\begin{array}{c} S_{k}^{3}=\{x_{i_{k}}^{3}={\left[W_{i_{k}},H_{i_{k}},E_{i_{k}}\right]}^{⊺}:i_{k}\in \Phi_{k}\},\;1\leq k\leq K. \end{array}$$

Fig 4 shows that the second cluster is the most expected for 3-dim clustering obviously because of three characteristics: low error rate of decisions (samll ERD), high workload (large NNoD) and stable information processing (good Hurst exponent). The distances between clusters in 3-dim feature space are greater than their counterparts in 2-dim feature space and separation of clusters is much more clear, as shown in Fig 4. By comparison with Figs 3 and 4, we will find that a higher-dimensional leads to a better clustering result, which will be proved in the following analysis.

## Assessing the attention qualities by statistical inference

### Comparison of 2-dim and 3-dim clustering

#### Centroid

According to the data collected by the NTT system for the ATC students, the centroids for the clusters are obtained, see Table 2. The centroids are computed by the following expressions:

$$\begin{array}{cc} \mu (S_{k}^{2}) & =\frac{1}{N_{k}}\sum_{x_{i_{k}}\in S_{k}^{2}}x_{i_{k}}=\frac{1}{N_{k}}\sum_{i_{k}=1}^{N_{k}}{\left[H_{i_{k}},W_{i_{k}}\right]}^{⊺}, \end{array}$$ (34)

$$\begin{array}{cc} \mu (S_{k}^{3}) & =\frac{1}{N_{k}}\sum_{x_{i_{k}}\in S_{k}^{3}}x_{i_{k}}=\frac{1}{N_{k}}\sum_{i_{k}=1}^{N_{k}}{\left[H_{i_{k}},W_{i_{k}},E_{i_{k}}\right]}^{⊺}. \end{array}$$ (35)

It clearly shows that the clusters respectively possess different centroids, which demonstrates the validation of the clustering algorithm. The centroids hold the role of the most representative points of each cluster.

**Table 2:** Centroids of 2-dim and 3-dim clusters.

Cluster $S_{k}^{2}$	Centroid $\mu \left(S_{k}^{2}\right)$	Cluster $S_{k}^{3}$	Centroid $\mu \left(S_{k}^{3}\right)$
$S_{1}^{2}$	[0.1771, 0.6390]^⊺^	$S_{1}^{3}$	[0.2714, 0.6032, 0.0510]^⊺^
$S_{2}^{2}$	[0.3455, 0.5530]^⊺^	$S_{2}^{3}$	[0.5789, 0.6675, 0.0680]^⊺^
$S_{3}^{2}$	[0.4571, 0.7216]^⊺^	$S_{3}^{3}$	[0.3980, 0.6683, 0.5880]^⊺^
$S_{4}^{2}$	[0.7244, 0.6335]^⊺^	$S_{4}^{3}$	[0.6590, 0.6490, 0.8619]^⊺^

#### Euclidean distance

**Fig 5:**
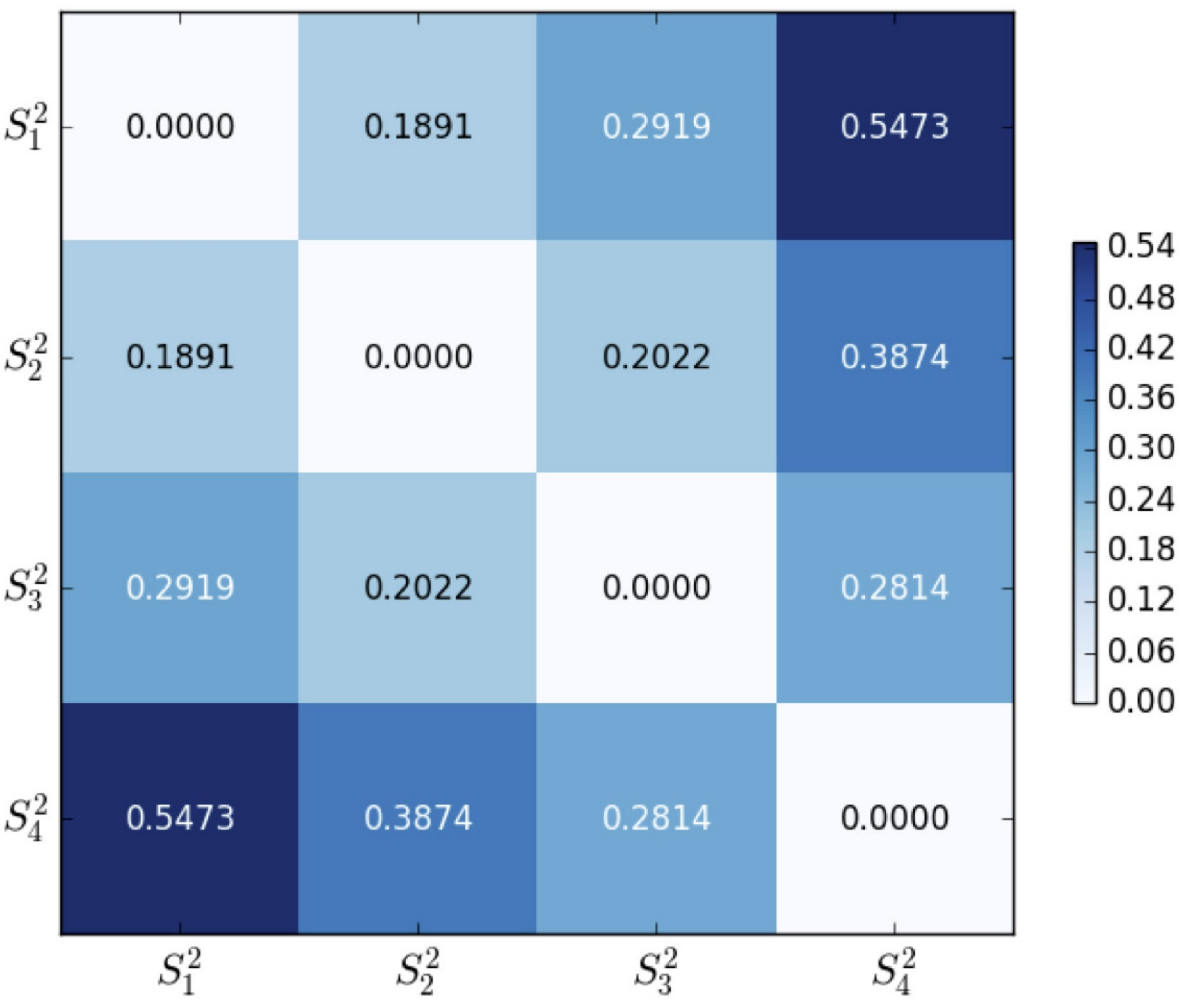
2-dim Euclidean distances of clusters ${\left\{S_{k}^{2}\right\}}_{k=1}^{4}$.

**Fig 6:**
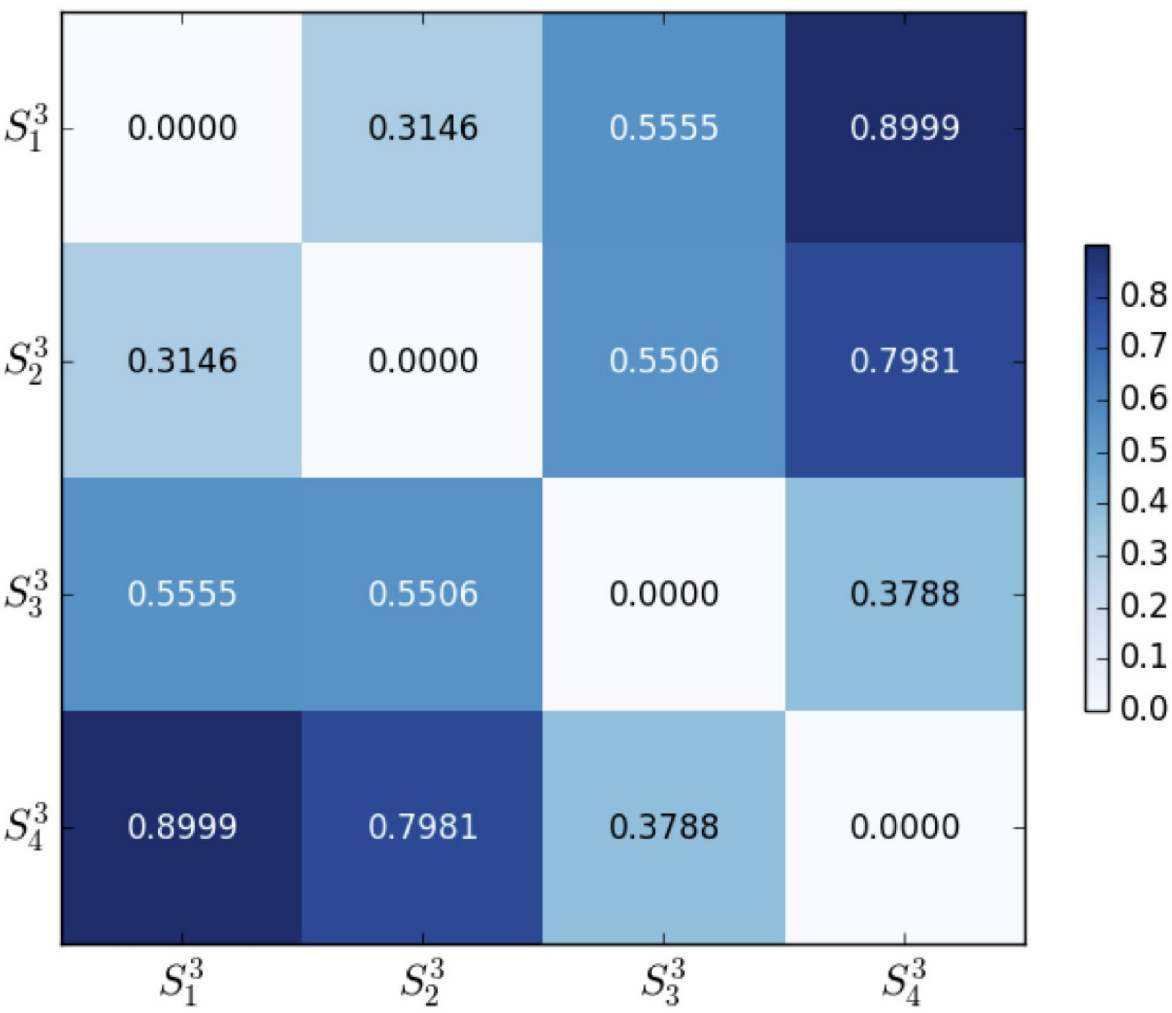
Euclidean distance of 3-dim clusters ${\left\{S_{k}^{3}\right\}}_{k=1}^{4}$.

Figs 5 and 6 are two heat-maps about the matrix of Euclidean distance ${\mathrm{dist}}_{E}\left(S_{k}^{n},S_{i}^{n}\right)$ between every two centroids by (18). The distance ranges for 2-dim and 3-dim clusters are $\left[0,\sqrt{2}\;\right]$ and $\left[0,\sqrt{3}\;\right]$ respectively. The Euclidean distance gives an intuitive clustering result. By comparison, we find that 3-dim clustering separates the sample space with more prominent distinctness. If the sample space is better partitioned into four clusters, then more distinct characteristics will be obtained. Consequently, clustering on a higher dimensional sample space could represent more profound IF hidden in the participants.

#### Second order statistics

For each cluster, either 2-dim or 3-dim clustering, the values of second order statistics are satisfactory for statistical inference:

- Table 3 shows that the variances and standard deviations of 2-dim and 3-dim clusters are small by Eq (20);
- Tables 4 and 5 show that the covariance matrices are diagonally dominant for each 2-dim cluster $S_{k}^{2}$ and 3-dim cluster $S_{k}^{3}$.

**Table 3:** Variances and standard deviations of clusters: 2-dim vs. 3-dim.

$S_{k}^{2}$	${\left[\sigma (S_{k}^{2})\right]}^{2}$	$\sigma (S_{k}^{2})$	$\frac{1}{2}\sigma \left(S_{k}^{2}\right)$	$S_{k}^{3}$	${\left[\sigma (S_{k}^{3})\right]}^{2}$	$\sigma (S_{k}^{3})$	$\frac{1}{3}\sigma \left(S_{k}^{3}\right)$
$S_{1}^{2}$	0.0595	0.2439	0.1220	$S_{1}^{3}$	0.0597	0.2443	0.0814
$S_{2}^{2}$	0.0150	0.1225	0.0613	$S_{2}^{3}$	0.0799	0.2867	0.0956
$S_{3}^{2}$	0.0223	0.1493	0.0747	$S_{3}^{3}$	0.0223	0.1526	0.0509
$S_{4}^{2}$	0.0118	0.1086	0.0542	$S_{4}^{3}$	0.0169	0.1300	0.0433

**Table 4:** Covariance matrix and eigenvalues for 2-dim clusters.

Cluster $S_{k}^{2}$	Covariance Matrix $\Sigma_{k}^{2}$	Eigenvalues $\text{Eig}(\Sigma_{k}^{2})$
$S_{1}^{2}$	$\left[\begin{array}{cc} +0.0076 & +0.0005\\ +0.0005 & +0.0047 \end{array}\right]$	{0.0076, 0.0046}
$S_{2}^{2}$	$\left[\begin{array}{cc} +0.0047 & -0.0007\\ -0.0007 & +0.0043 \end{array}\right]$	{0.0053, 0.0038}
$S_{3}^{2}$	$\left[\begin{array}{cc} +0.0049 & -0.0008\\ -0.0008 & +0.0045 \end{array}\right]$	{0.0055, 0.0039}
$S_{4}^{2}$	$\left[\begin{array}{cc} +0.0106 & +0.0029\\ +0.0029 & +0.0093 \end{array}\right]$	{0.0129, 0.0070}

**Table 5:** 3-dim clusters, covariance matrix and eigenvalues.

Cluster $S_{k}^{3}$	Covariance Matrix $\Sigma_{k}^{3}$	eigenvalues $\text{Eig}(\Sigma_{k}^{3})$
$S_{1}^{3}$	$\left[\begin{array}{ccc} +0.0105 & -0.0015 & -0.0009\\ -0.0015 & +0.0073 & +0.0019\\ -0.0009 & +0.0019 & +0.0039 \end{array}\right]$	{0.0114, 0.0073, 0.0030}
$S_{2}^{3}$	$\left[\begin{array}{ccc} +0.0208 & -0.0015 & +0.0008\\ -0.0015 & +0.0123 & -0.0005\\ +0.0008 & -0.0005 & +0.0061 \end{array}\right]$	{0.0211, 0.0121, 0.0060}
$S_{3}^{3}$	$\left[\begin{array}{ccc} +0.0065 & +0.0035 & +0.0019\\ +0.0035 & +0.0081 & +0.0044\\ +0.0019 & +0.0044 & +0.0101 \end{array}\right]$	{0.0152, 0.0062, 0.0032}
$S_{4}^{3}$	$\left[\begin{array}{ccc} +0.0092 & -0.0011 & +0.0025\\ -0.0011 & +0.0027 & +0.0046\\ +0.0025 & +0.0046 & +0.0112 \end{array}\right]$	{0.0003, 0.0090, 0.0139}

Obviously, this means good inter-cluster statistical characteristics. In other words, the validation of clustering algorithm has been proved once again.

Once two clusters are considered at the same time, we can find the intra-cluster characteristics with the covariance matrix by (22). Covariance matrix represents the relevance among different features and its eigenvalues indicate the contribution of the corresponding feature to the characteristic of an individual (participant of NTT).

With the help of Table 6 for the 2-dim clustering, we find that the features are weakly dependent due to the small correlation coefficients Cr_*ij*,*i*≠*j*_ < 0.3 by (23). However, Table 6 also shows that for the 3-dim clustering the dependency among three features is higher with the similar calculation for the correlation coefficients. In other words, the analysis explored on the 3-dim feature space is more complex than its counterpart in 2-dim space. The higher the dependency of the features, the stronger the cross-impact of the IQ is. Intuitively, the attention span, stability of attention, distribution-shift of attention may interplay in some sense. Fortunately, both correlation coefficient of these clusters own a weak dependency among different features, i.e., the features of the IQ corresponding to an excellent participant are nearly independent.

**Table 6:** Pairwise correlation coefficient of *W*, *H* and *E* for specified clusters.

Cluster	$\mathrm{Cr}[W\left(S_{k}^{2}\right),H\left(S_{k}^{2}\right)]\}$	Cluster	$\mathrm{Cr}[W\left(S_{k}^{3}\right),H\left(S_{k}^{3}\right),E\left(S_{k}^{3}\right)]$
$S_{1}^{2}$	$\left[\begin{array}{cc} +1.0000 & +0.0776\\ +0.0776 & +1.0000 \end{array}\right]$	$S_{1}^{3}$	$\left[\begin{array}{ccc} +1.0000 & -0.1672 & -0.1345\\ -0.1672 & +1.0000 & +0.3577\\ -0.1345 & +0.3577 & +1.0000 \end{array}\right]$
$S_{2}^{2}$	$\left[\begin{array}{cc} +1.0000 & -0.1631\\ -0.1631 & +1.0000 \end{array}\right]$	$S_{2}^{3}$	$\left[\begin{array}{ccc} +1.0000 & -0.0925 & +0.0681\\ -0.0925 & +1.0000 & -0.0529\\ +0.0681 & -0.0529 & +1.0000 \end{array}\right]$
$S_{3}^{2}$	$\left[\begin{array}{cc} +1.0000 & -0.1616\\ -0.1616 & +1.0000 \end{array}\right]$	$S_{3}^{3}$	$\left[\begin{array}{ccc} +1.0000 & +0.4913 & +0.2362\\ +0.4913 & +1.0000 & +0.4871\\ +0.2362 & +0.4871 & +1.0000 \end{array}\right]$
$S_{4}^{2}$	$\left[\begin{array}{cc} +1.0000 & +0.2932\\ +0.2932 & +1.0000 \end{array}\right]$	$S_{4}^{3}$	$\left[\begin{array}{ccc} +1.0000 & -0.2288 & +0.2447\\ -0.2288 & +1.0000 & +0.8244\\ +0.2447 & +0.8244 & +1.0000 \end{array}\right]$

#### TrLogDet distance

**Fig 7:**
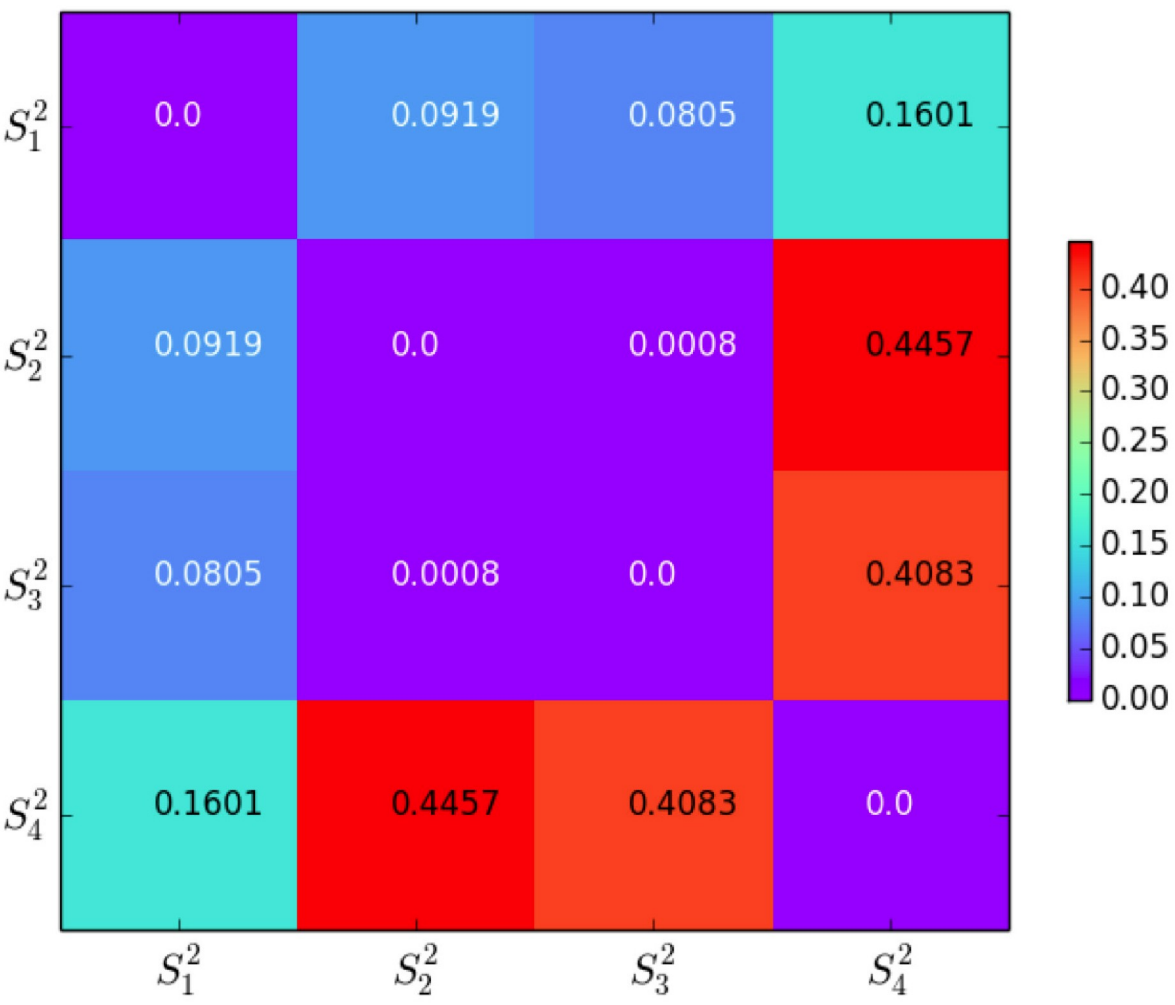
2-dim clusters and TrLogDet distance per dimension.

**Fig 8:**
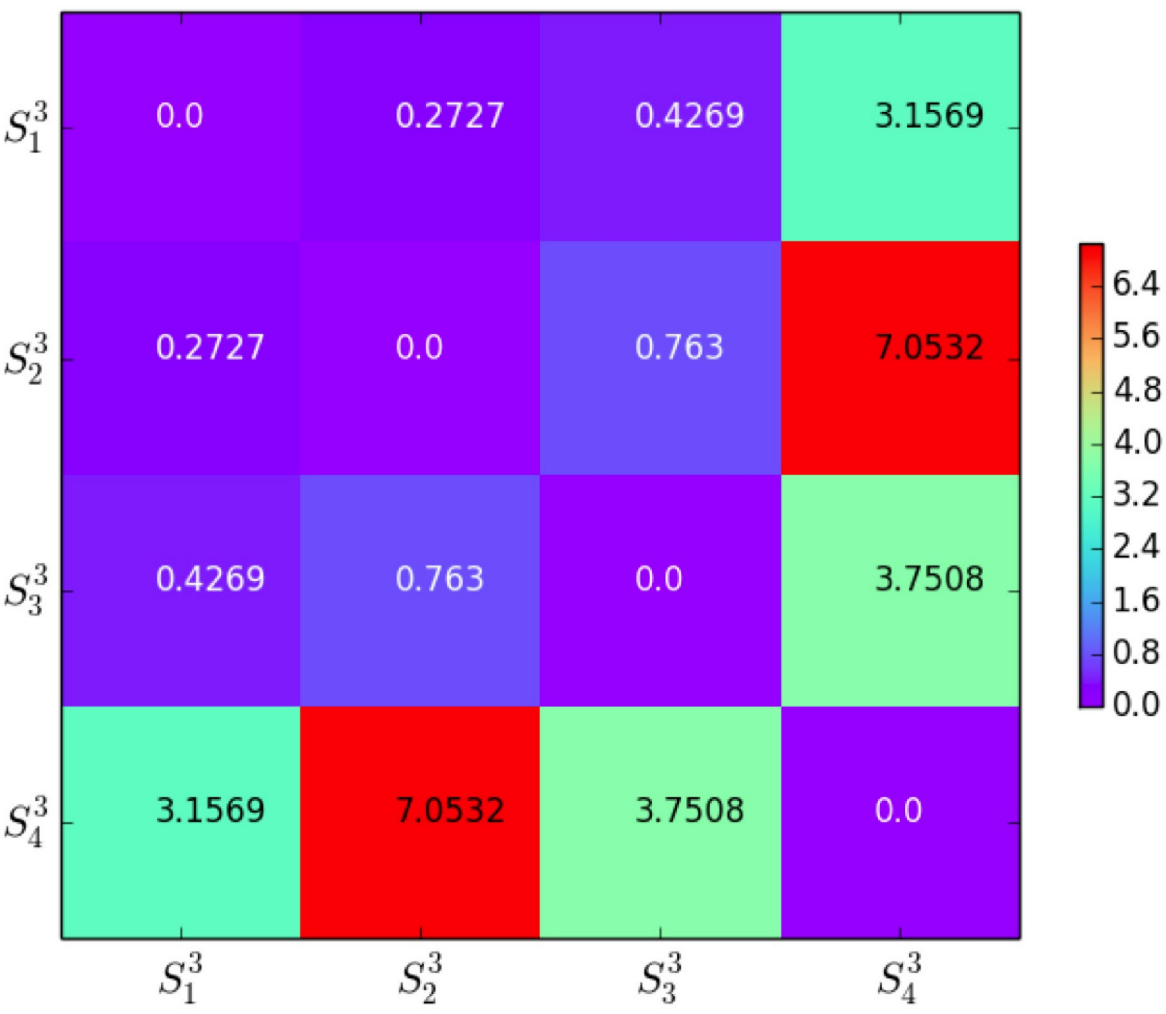
3-dim clusters and TrLogDet distance per dimension.

The TrLogDet distance ${\mathrm{dist}}_{T}\left(S_{k}^{n},S_{i}^{n}\right)$ for each two clusters are computed according to Eq (25), see Figs 7 and 8. We find that TrLogDet values for the 2-dim clusters are smaller than their counterparts in 3-dim space. For 2-dim clustering, the clusters have similar profiles where the samples locate within a neighborhood. By contrast, for 3-dim clustering, the samples spread in a wide range. Although the clustering in 3-dim sample space has higher complexity and the assessments for 3-dim objects is more complicated, the 3-dim result is more persuasive in general since it contains more information.

#### Information theoretic metric

The information theoretic metric (ITM) is calculated via (26), see Table 7. The ITMs for the four 2-dim clusters have no significant difference, which means their profiles are similar. However, for the 3-dim clusters, the difference of ITMs are much larger than those in 2-dim space. This implies two facts:

- the feature points can be distinguished well with ITM if the dimension can be set up properly;
- the performance of 3-dim clustering is better than 2-dim clustering since more informaton is involved in for the purpose of distinguishing feature points.

**Table 7:** ITM per dimension for 2-dim and 3-dim clusters.

*k*	1	2	3	4
$\rho (S_{k}^{2})$	8.2740	8.8435	8.7624	7.3384
$\rho (S_{k}^{3})$	12.2218	10.4285	12.0250	14.2618

#### Sum of squared errors

According to Eq (27) for SSE, we obtain Table 8. For the SSE of *n*-dim clustering, a small value of SSE per sample reflects satisfactory clustering outcome for a given *K*. A good choice of *K* depends on the characteristics of the data set, experiences, and objective of the data analysis. In our study, we set *K* = 4.

**Table 8:** SSE for 2-dim and 3-dim clusters.

*k*	1	2	3	4	Amounts
$\mathrm{SSE}(S_{k}^{2})$	0.1063	0.0104	0.0200	0.0032	0.1399
$\mathrm{SSE}\left(S_{k}^{2}\right)/|S_{k}^{2}|$	0.0034	0.0002	0.0005	0.0001	0.0042
$\mathrm{SSE}(S_{k}^{3})$	0.2563	0.3131	0.0055	0.0020	0.6291
$\mathrm{SSE}\left(S_{k}^{3}\right)/|S_{k}^{3}|$	0.0034	0.0061	0.0005	0.0003	0.0103

### Clusters and performances of attention

#### 2-dim clusters of features [*W*, *H*]^⊺^

**Fig 9:**
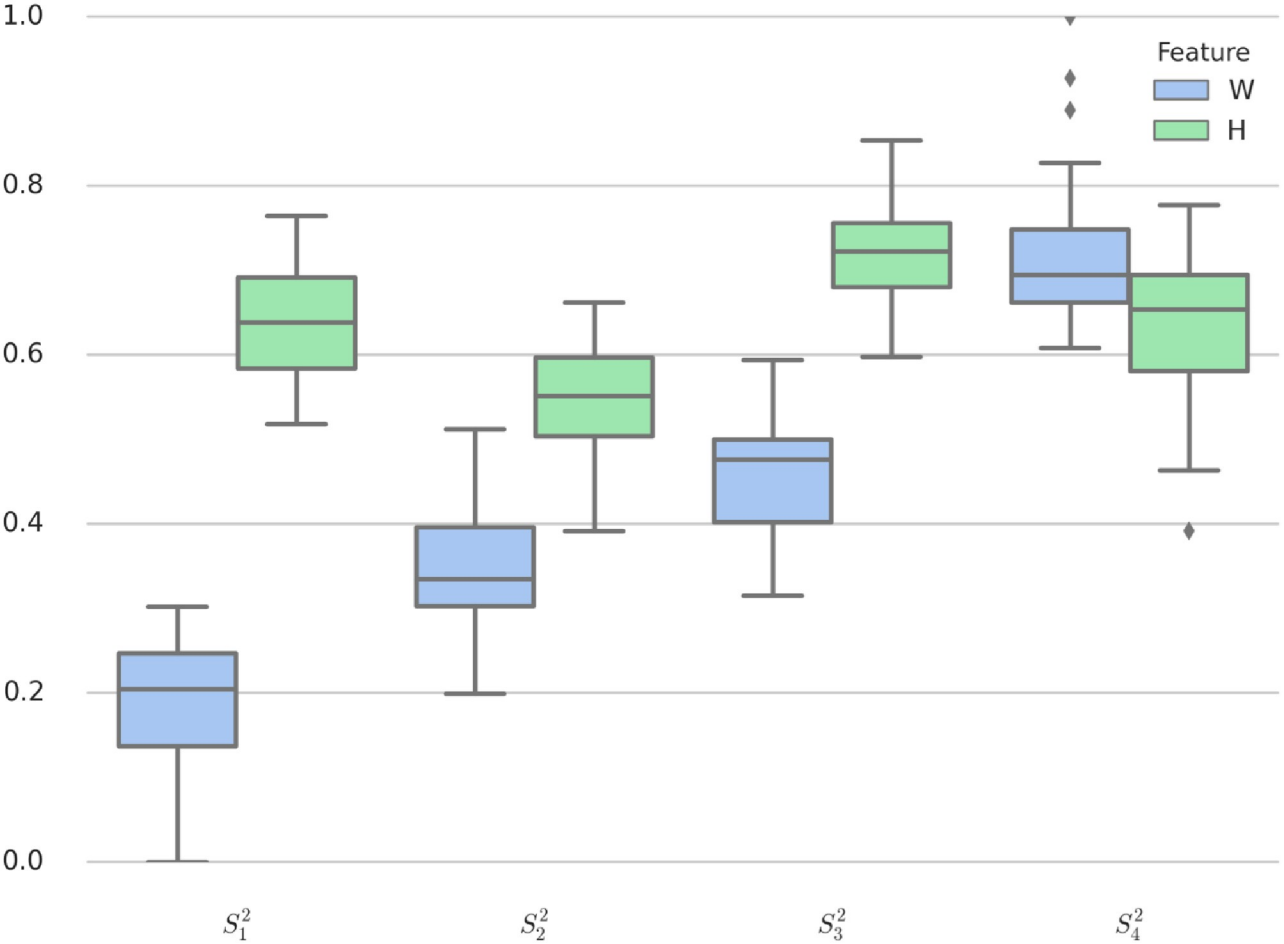
Box-plot of 2-dim clustering data.

Fig 3 illustrates the distinct clusters with *K*-means algorithm and *K* = 4. It is clear that the centroids of the four clusters are separated reasonably. For the 2-dim clusters demonstrated in Fig 3, the corresponding box-plot is shown in Fig 9. With the four levels (high, moderate, low and bottom) mentioned above, we now propose some interpretations as following:

- $S_{1}^{2}$ with bottom *W* and moderate *H*
  - The average *W* is the smallest among the four clusters, which indicates that the participants’ reaction is very slow and they spend much time to make a decision. Experimentally, the process for making the symbolic decision *y*_*i*_ ∈ {*Y*, *N*, *I*} is slow in the sense of statistics. Psychologically, it implies that the attention span of the participants is small.
  - The average *H* is about 0.64 which shows a good positive correlation in the sense of long term time [31]. Therefore, the attention stability of the participants is acceptable.
  - This type of controllers are not appropriate for busy airports with large traffic flow. On the contrary, only small airports with fewer flights could be considered for these controllers. For the sake of low operation risk, the participants should be allocated with low workload.
- $S_{2}^{2}$ with low *W* and bottom *H*
  - *H* floats up and down at 0.5, which indicates that the participants are most unstable during decision-making since *H* = 0.5 means strong randomness and poor correlation [31]. Therefore, the attention of the participants is not stable.
  - Low *W* shows that more time is needed for decision-making, thus the attention span is small.
  - The participants included in $S_{2}^{2}$ are not appropriate for being selected as controllers since neither the attention span nor the attention stability is good in the sense of controlling the operation risk.
- $S_{3}^{2}$ with moderate *W* and high *H*
  - For a given period, the NoD of participants in the 3rd cluster is stable. *H* above 0.6 shows that their response sequences are of the following characteristics: better long-range correlation and respondent continuity as well as positive correlation.
  - Moderate *W* indicates an ordinary level of decision-making of participants and good attention span.
  - Samples in cluster $S_{3}^{2}$ and cluster $S_{2}^{2}$ are at opposite positions and also with opposite characteristics. It proves that the clustering algorithm is capable of partitioning the samples with significant differences.
  - $S_{3}^{2}$ includes the best participants separated automatically via 2-dim clustering process.
- $S_{4}^{2}$ with high *W* and low *H*
  - High *W* means high attention span while low *H* means low attention stability.
  - The corresponding participants could work with those in $S_{3}^{2}$ when the workload of controllers is large in the cases of peak air traffic flow at busy airports.

#### 3-dim clusters of features [*W*, *H*, *E*]^⊺^

**Fig 10:**
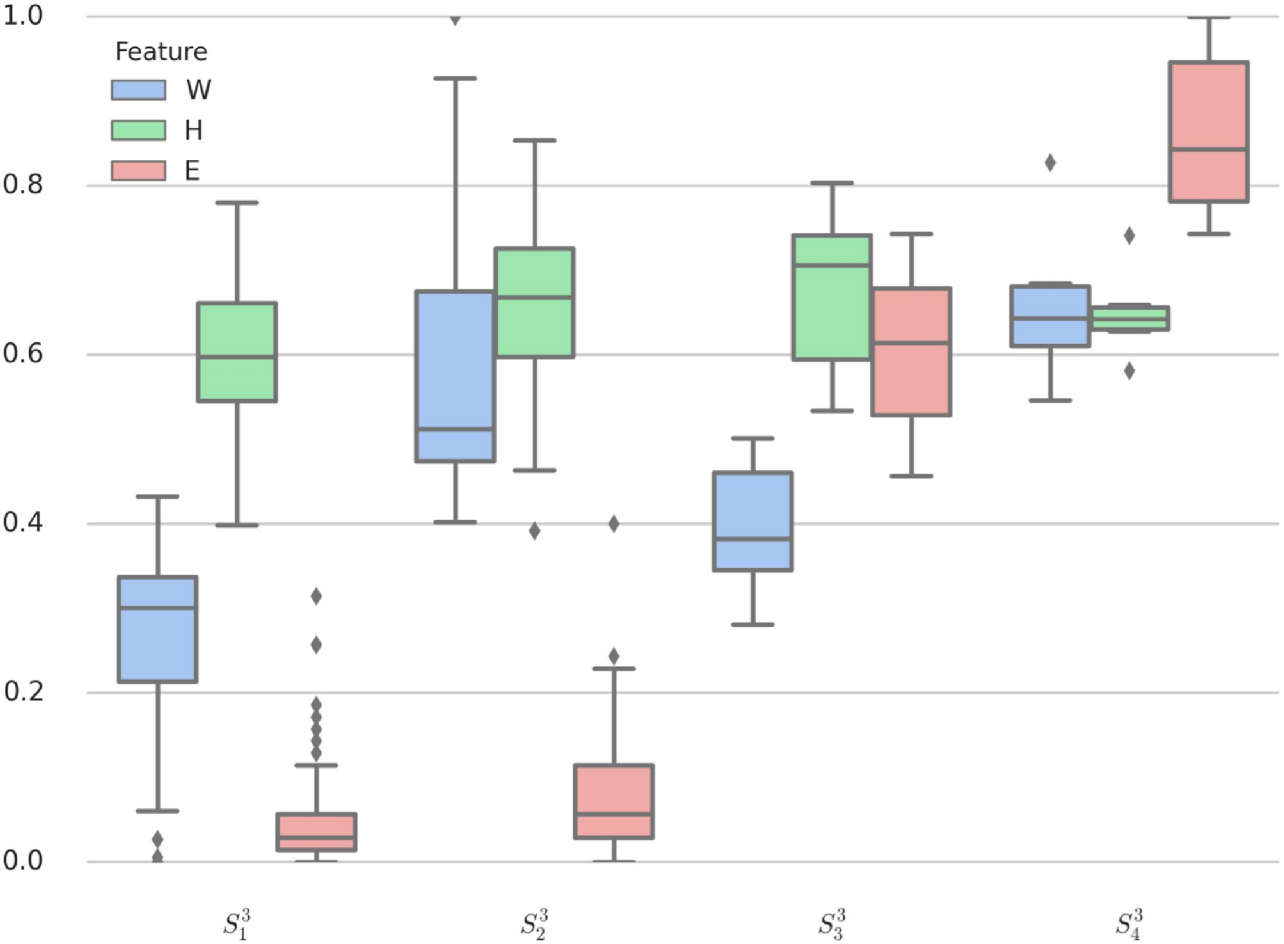
Box-plot of 3-dim clustering data.

Fig 4 illustrates the result of clustering via *K*-means algorithm with *K* = 4 for the 3-dim features $x_{k}^{3}={\left[W_{k},H_{k},E_{k}\right]}^{⊺}$ and the corresponding box-pot is given in Fig 10. It is clear that the centroids of the four clusters are separated more distinctly than those in 2-dim space.

For 3-dim clustering, the rank of *H* in Table 9 has no distinct difference because of *H* ∈ [0.6, 0.7] for four different clusters (which implies good positive long-range correlations and attention stability). We now give some interpretations for these clusters as follows:

- $S_{1}^{3}$ with bottom *W*, low *E* and poor *H*
  - The process of decision-making of participants in cluster $S_{1}^{3}$ needs a long time to make decisions, thus the attention span must be small. Meanwhile, a sufficient thinking time will lead to high accuracy or equivalently low *E*.
  - The values of *H* spread in a wide range which covers the critical point *H* = 0.5, thus their long-range correlation is relatively poor. We can infer that the occurrence of mistakes fluctuates rapidly and randomly. In consequence, the attention stability is poor.
- $S_{2}^{3}$ with moderate *W*, low *E* and a wide range of *H*
  - Moderate *W* of the participants in cluster $S_{2}^{3}$ indicates a moderate level of information processing and attention span. Particularly, 25% of the participants in $S_{2}^{3}$ have excellent attention span since they have large *W* values when compared with other clusters.
  - Low *E* shows the high accuracy during decision-making. Therefore, the distribution-shift of attention is good and acceptable.
  - The value of *H* has a wide range, which implies the long-range correlation hidden in the response sequence is complex. However, for the participants in $S_{2}^{3}$, the box-plot also shows that more 70% of them have good attention stability since *H* ≥ 0.6 and 25% of them have excellent attention stability since *H* ≥ 0.7.
  - In general, $S_{2}^{3}$ is the best cluster for 3-dim clustering and the participants of interest can be selected objectively since the IQ are the best.
- $S_{3}^{3}$ with low *W*, moderate *E* and high *H*
  - About 75% of the participants have good attention stability since *H* > 0.6 and 25% of them have moderate *H*. Moreover, it is clear that the *W* is low and *E* is high since *W* ≤ 0.5 and *E* ≥ 50%.
  - Obviously, the participants characterized by $S_{3}^{3}$ have poor work performances: low attention span, low distribution-shift of attention and strong stability. In other words, they response slowly, make decisions with high error rate, and it is difficult for them to improve their work efficiency and correctness of decisions.
- $S_{4}^{3}$ with high *W*, low *H*, and high *E*
  - Both the *W* and the *H* are moderate, thus the attention span and stability of the participants in $S_{4}^{3}$ are acceptable.
  - The distribution-shift of attention is very poor since *E* > 70% for each participant and *E* ≈ 100% for participants. This implies that the participants should be re-evaluated seriously and they may not be selected as air traffic controllers.

**Table 9:** Rank the IQ for 3-dim clustering result.

Cluster	Span	Stability	distribution-shift
$S_{1}^{3}$	Bottom	Bottom	**High**
$S_{2}^{3}$	Moderate	Moderate	Moderate
$S_{3}^{3}$	Low	**High**	Low
$S_{4}^{3}$	**High**	Low	Bottom

### Levels of intrinsic qualities for ATC students and controllers

According to the results of 2-dim and 3-dim clustering analysis that are shown in Figs 3 and 4 respectively, we invited five experts of ATC with more than 10 years work experiences to evaluate attention span, attention stability and distribution-shift of attention of the controllers (grade from 1 to 9 points). The higher the score is, the better the attention features are. In the end, we get three intervals: 1 ∼ 3 points, 4 ∼ 6 points and 7 ∼ 9 points, see Table 10.

**Table 10:** Expert evaluation on IF influencing VAP.

IF of Attention	IQ	1 ∼ 3 Points	4 ∼ 6 Points	7 ∼ 9 Points
Span	*W*	Narrow	Medium	Wide
Stability	*H*	Bad	Medium	Good
Distribution-shift	*E*	Slow & Inaccurate	Medium	Rapid & Exact

Based on the assessment of IQ for 2-dim and 3-dim clusters, the ATC students can be divided into four classes, from which the classification could be generalized to the assessment of air traffic controllers, see Table 11. Consequently, we have

**Table 11:** Assessment of air traffic controllers’ IQ influencing VAP: A generalization.

Level	Span	Stability	Distribution-Shift	Volatility
1	Wide	Good	Rapid & Exact	Quasi-Stationary
2	Wide	Good	Slow & Inaccurate	Small-Amplitude
3	Narrow	Bad	Rapid & Exact	Large-Amplitude
4	Narrow	Bad	Slow & Inaccurate	Small-Amplitude

- *Level-1* air traffic controllers—Concentrating attention rapidly, distributing attention reasonably, and finishing the specific task with high accuracy subject to high workload. It is feasible to allocate complex air traffic control tasks for this kind of controllers.
- *Level-2* air traffic controllers: Processing information swiftly and stably with high error rate. In other words, they may suffer great difficulties if multi-tasks or the task with multi-objectives are concerned. This kind of controllers may make lots of mistakes due to high air traffic flow. Their low performance of distribution-shift of attention might cause omission of traffic conflicts, which may increase the operation risk.
- *Level-3* air traffic controllers: The distribution-shift of attention is swift. However, neither the attention span is sufficient nor the attention stability is acceptable. Apparently, the “error/mistake”,“forgetting” and “omission” phenomenon appear frequently if the controllers’ workload is high. As a suggestion, it would be better for this kind of controllers to work in the period of an average air traffic flow intensity or in a small airport with low air traffic flow intensity.
- *Level-4* air traffic controllers: Characterized by a non-timely reaction and high probability of making mistakes. Generally, the inferior IQ of Level-4 controllers may cause serious operation risk.

## Conclusions

### Key results

Aviation safety is closely related with the IF influencing the VAP of air traffic controllers. However, there is a lack of mathematical descriptions and effective analysis of IF based on observable data. We presented the IQ—a mathematical representation for the IF—and constructed the interested 2-dim and 3-dim feature spaces with the computational parameters NNoD, Hurst exponent and ERD for characterizing the response sequences. The *K*-means clustering algorithm is applied for the IQ and the systematic assessment is performed for the clusters in 2-dim and 3-dim through statistical inference through computing and comparing the geometric metrics and statistics defined.

By the NTT system and the response sequence collected from 143 ATC students, the statistical analysis for the clusters obtained demonstrates some interesting results:

- The data set has been well divided into four clusters which coincides with the practical observations for the air traffic controllers.
- The eigen-features of the clusters and the correlation coefficients for the directly measurable IQ, i.e., NNoD, Hurst exponent and ERD, show that each cluster has its own eigen-features and manifests its unique characteristics, thus the corresponding controllers or professionals may have specific operation risk.
- The participants can be divided into four levels: Excellent, Good, Medium, Unqualified, which implies:
  - the potential rules for selecting controllers for specific tasks in different air traffic flow intensity in order to satisfy the demand for ensuring safe operations in the civil aviation;
  - the controllers in different clusters should be allocated with different workload when the type of airports and the air traffic flow intensity are considered.

In the sense of methodology, our work illustrates a novel chain of logic for analyzing and reducing the operation risk in civil aviation: professionals → VAP → IF → IQ → clustering → statistical inference → IQ levels → human errors. We could hope that the method developed in this work will be helpful for selecting professionals and allocating workload more reasonably subject to operation risk allowed in lots of fields.

### Future work

Generally, there are plenty of forms of human factors which have impact on aviation safety. The “error/mistake”, “forgetting”, “omission” are three kinds of top factors which can be captured by NTT system. Statistical inference itself shows that we should design experiments for collecting data carefully and objectively as possible as we can. In future, we will pay more attention to the following aspects:

- Explore new IF influencing VAP which can be captured by the NTT system. We just take three intrinsic factors and the corresponding IQ in this work. It is still an open problem that seeking measurable VAP and their representations (IQ).
- Invite left-handers to do the tests with NTT system. Currently, all of the ATC students invited are right-handers. Thus, the visual attention is mainly focused on the left brain in the sense of neurophysiology and cognitive psychology. In the future, we will also collect the data from the left-handers.
- Increase the number of participants, from which we can utilize the statistical theory of a large number of samples. The more participants, the more confidential and convincing the levels of IQ for air traffic controllers.
- Determine the parameter *K* automatically. In our paper, the parameter *K* for clustering is set as *K* = 4 according to our experiences and knowledge of classifying air traffic controllers, which has potential impact on the statistical inference. In the future, we will design algorithms to select the parameter *K* automatically and find a trade-off between the complexity of clustering algorithm and difficulty of interpreting the clusters obtained subject to the facts and requirements of ATC.

## Supporting information

S1 Appendix Data of NTT. The data used in this paper is uploaded. For more questions, please contact with the first author Dr. Jing-Qiang Li (jingqiang_li@foxmail.com).
(XLSX)
